# Brain structure characteristics in children with attention-deficit/hyperactivity disorder elucidated using traveling-subject harmonization

**DOI:** 10.1038/s41380-025-03142-6

**Published:** 2025-08-08

**Authors:** Qiulu Shou, Masatoshi Yamashita, Yoshiyuki Hirano, Akiko Yao, Min Li, Yide Wang, Yoko Kato, Tokiko Yoshida, Koji Matsumoto, Tetsuya Tsujikawa, Hidehiko Okazawa, Akemi Tomoda, Kuriko Kagitani-Shimono, Yoshifumi Mizuno

**Affiliations:** 1 Research Centre for Child Mental Development, University of Fukui, Fukui, Japan; 2 United Graduate School of Child Development, Osaka University, Kanazawa University, Hamamatsu University School of Medicine, Chiba University and University of Fukui, Osaka, Japan; 3 Research Centre for Child Mental Development, Chiba University, Chiba, Japan; 4 Department of Radiology, Chiba University Hospital, Chiba, Japan; 5 Department of Radiology, Faculty of Medical Sciences, University of Fukui, Fukui, Japan; 6 Biomedical Imaging Research Centre, University of Fukui, Fukui, Japan; 7 Department of Child and Adolescent Psychological Medicine, University of Fukui Hospital, Fukui, Japan; 8 Molecular Research Centre for Children’s Mental Development, Osaka University Graduate School of Medicine, Osaka, Japan; 9 Department of Pediatrics, Osaka University Graduate School of Medicine, Osaka, Japan

**Keywords:** Neuroscience, ADHD

## Abstract

Brain imaging studies for attention-deficit/hyperactivity disorder (ADHD) have not always yielded consistent findings, potentially owing to measurement bias in magnetic resonance imaging (MRI) scanners. This study aimed to elucidate the structural brain characteristics in children with ADHD by addressing measurement bias in multi-site MRI data using the harmonization method, traveling-subject (TS) approach. The MRI data of 14 traveling subjects, 178 typically developing (TD) children, and 116 children with ADHD were collected from multiple sites. The TS method and ComBat were used to correct for measurement bias. Gray matter volumes were estimated using FreeSurfer, and the ADHD and TD groups were compared using mixed-effect models. Compared to raw data, the TS method significantly reduced measurement bias while maintaining sampling bias. In contrast, ComBat effectively reduced measurement bias but also significantly decreased sampling bias. TS-corrected data showed decreased brain volumes in the frontotemporal regions in the ADHD group compared to the TD group. Specifically, significant volumetric reductions were found in the right middle temporal gyrus in children with ADHD (TS-corrected data: *β* = −0.255, FDR [family discovery rate] *p* = 0.001). These results demonstrate that the TS method effectively reduces measurement bias across MRI scanners, ensuring reliable findings in multi-site studies. The observed frontotemporal volume reductions in ADHD, especially in the right middle temporal gyrus, highlight the reliability of findings obtained with TS correction.

## Introduction

Attention deficit hyperactivity disorder (ADHD) is a common disorder most frequently diagnosed in children and adolescents, with a prevalence of over 5% [1, 2]. It is characterized by inattention, overactivity, or impulsiveness symptoms that are age-inappropriate, persistent, and pervasive [1]. In the long term, ADHD is associated with a significant risk of educational failure, interpersonal problems, mental illness, and delinquency [3], placing a substantial burden on families as well as on health, social care, and criminal justice systems [4]. Therefore, it is important to clarify the neural basis of ADHD and establish effective early assessments and interventions.

Although many brain imaging studies have been conducted to elucidate the pathology of ADHD and abnormalities in brain structure in children with ADHD have been reported, these studies have not always yielded consistent findings [5]. Some studies have found that patients with ADHD showed decreased grey matter volume in the extensive parts of the cortex, including the dorsolateral prefrontal cortex, anterior cingulate cortex, inferior parietal lobule [6–12], and subcortical areas, including the amygdala, caudate nucleus, hippocampus, and putamen [7, 9], compared with control subjects. By contrast, another study found no significant reduction in gray matter volume after multiple comparison corrections [13]. Other studies on patients with ADHD have found larger gray matter volumes in the dorsolateral prefrontal, temporal, and intra-calcarine cortices, parietal lobule [13–15], left caudate nucleus, and putamen [10].

As described above, ADHD-related neuroimaging studies have provided conflicting evidence, implying that the neurobiological mechanisms underlying ADHD lack clarity, possibly because of small sample sizes, differences in magnetic resonance imaging (MRI) scanners, selection of participants, and so on [5]. To control for the MRI machines, previous studies have used the mixed-effect model to control for MRI machines as a random effect or used ComBat harmonization [16] to control site and MRI differences in large samples through meta-analyses and multi-institutional studies [17]. However, there are two problems with such methods: (1) it is difficult to extract all MRI differences and correct for measurement bias, and (2) it is possible to reduce sampling bias incorrectly [17]. To solve these problems, a new correction approach, the traveling-subject (TS) method, has been developed [17, 18]. The TS method is an innovative approach for correcting differences between MRI machines. Using the TS method, sampling bias and individual differences can be controlled for the same participant using MRI scans from multiple institutions, and the regression model only extracts the measurement bias between models, enabling more accurate data correction [17]. A previous study provided evidence for the applicability of the TS method to the structural brain [18].

Therefore, in this study, we first examine the validity of the TS method by computing the reliability of the harmonized brain structure in the TS dataset. Next, we apply the TS method to an independent dataset, including 178 children with typical development (TD) and 116 children with ADHD from hospitals of the University of Fukui, Osaka University, and Chiba University. Then we compare the measurement and sampling biases among the TS-corrected data, ComBat-corrected data, and raw data to assess the applicability of the TS method to the independent structural brain dataset. Though a previous study provided evidence of the applicability of the TS method in structural brain data [18], few studies applied the TS method to an independent dataset of brain structures. Moreover, this study will analyze the differences in brain structural characteristics between children with ADHD and children with TD and compare the results of TS-corrected, ComBat-corrected, and raw data. This is the first study to apply the TS method to correct for measurement bias from different MRI scanners in multi-site brain structure data, and the methods of accurate removal of measurement bias discovered in this study may help ensure the reproducibility of results from ADHD brain structure studies.

## Subjects and methods

### Participants

Fourteen healthy TS participants (female = 7, age = 31.71 ± 8.20 years, right-handedness = 13) underwent MRI scans at four different machines (two at the University of Fukui, one at Osaka University, and one at Chiba University) over a three-month period. The study used the TS dataset from the Child Developmental MRI (CDM) project [5] to address measurement bias in each MRI machine. Participants with ADHD were recruited from hospitals of the University of Fukui, Osaka University, and Chiba University in Japan. Children with TD were recruited from the local community and assessed to ensure that none of them had developmental delays, received any special support education, or had a history of epilepsy or other psychiatric disorders. Participants with ADHD fulfilled the diagnostic criteria for ADHD according to the Diagnostic and Statistical Manual of Mental Disorders Fifth Edition (DSM-5). Participants in the current study participated in the experiments from 2014 to 2022. None of the participants had a history of severe head trauma, neurological illness, or potential for hazards associated with MRI examinations (such as the presence of metal on the body surface or internal structures, pregnancy, claustrophobia, or fear of the dark). The demographic data of the participants with ADHD and TD in each MRI machine are summarized in Tables 1 and S1.

**Table 1:** Demographic data of the participants.

	TD(*N* = 178)	ADHD(*N* = 116)	Difference between TD and ADHD (*p*-value)
Age (years)	12.71 ± 2.80	10.27 ± 2.23	*p* < 0.001
Sex			
Male/Female(n)	119/59	108/8	*p* < 0.001
Handedness(n)			
Right/Left/Ambidextrous (n)	163/12/3	102/13/1	*p* > *0.05*
IQ	105.52 ± 11.14	95.28 ± 13.32	*p* < 0.001
ICV (cm^3^)	1391.82 ± 172.56	1382.03 ± 16.73	*p* > 0.05

*TD* typical development, *ADHD* attention-deficit/hyperactivity disorder, *n* number, *IQ* intelligence quotient, *ICV* intracranial volume.

### MRI data acquisition

Participants were scanned with T1-weighted imaging at the University of Fukui, Osaka University, or Chiba University using a 3T GE Signa PET/MR scanner (General Electric HealthCare, Chicago, Illinois, USA; University of Fukui), 3T GE Discovery MR750 scanner (General Electric HealthCare; University of Fukui or Chiba University), or 3T GE Signa Architect scanner (General Electric HealthCare; Osaka University). The scanning parameters are provided in Table S2.

### MRI analysis

The fully automated segmentation procedure implemented in FreeSurfer version 7.3.8 was used to estimate the gray matter volumes of the cortical and subcortical regions (http://surfer.nmr.mgh.harvard.edu/). The structural data were obtained using a standardized processing pipeline. The analysis used the Desikan-Killiany atlas for classifying cortical regions (68 brain regions) and for segmenting subcortical regions (14 brain regions, such as thalamus, caudate, putamen, pallidum, hippocampus, amygdala, and accumbens). Details of the segmentation method are provided by Fischl et al. [19].

### Harmonization methods

We followed the TS harmonization method reported by Yamashita et al. [5], which extends a general linear model harmonization using the TS dataset. Python was used to estimate the measurement bias of each MRI machine using the TS dataset and reduce measurement bias from the CDM dataset. We first utilized the TS dataset to calculate scanner differences using ridge regression. The model included dummy variables for both the 4 scanners and the 14 TS participants as follows:

$${{{{\rm{Brain\,structures}}}}={{{\rm{X}}}}}_{{{{\rm{m}}}}}{}^{{{{\rm{T}}}}}{{{\rm{m}}}}+{{{{\rm{X}}}}}_{{{{\rm{p}}}}}{}^{{{{\rm{T}}}}}{{{\rm{p}}}}+{{{\rm{e}}}}$$

Here, ***m*** signifies the measurement bias (4 machines × 1), and ***p*** signifies the TS participant factor (14 TS participants × 1).

There is no sampling bias in the TS participants, as participants across different MRI machines do not differ. The TS harmonization method only estimates variations between MRI scanners. Once we estimated the machine differences using the model above, we applied them to the CDM dataset to correct the measurement bias.

ComBat harmonization was also used to control measurement bias for comparison. ComBat was initially developed to correct the batch effect in genomics [20] and has recently been applied to MRI datasets [18]. ComBat corrects a type of multivariate dataset using an empirical Bayesian estimation approach and can be used to analyze datasets obtained using different scanning machines. In the current study, we used the module “neuroCombat” to correct structural brain data using Python [21]. We used ComBat harmonization in the TS dataset and CDM dataset individually. In the TS dataset, we included age, sex, and handedness as covariates for data correction. Whereas, in the CDM dataset, we included age, sex, handedness, and diagnosis (ADHD or TD) as covariates.

### Measurement and sampling biases of different harmonization methods

To quantitatively investigate the validity of different harmonization methods in structural brain data, we calculated measurement biases, sampling biases, and disorder factors, following recommendations from Yamashita et al. [17]. We estimate the measurement and sampling biases using the following model:

$${{{\rm{Brain\,structures}}}}={{{{\rm{X}}}}}_{{{{\rm{m}}}}}{}^{{{{\rm{T}}}}}{{{\rm{m}}}}+{{{{\rm{X}}}}}_{{{{\rm{s}}}}}{}^{{{{\rm{T}}}}}{{{\rm{s}}}}+{{{{\rm{X}}}}}_{{{{\rm{d}}}}}{}^{{{{\rm{T}}}}}{{{\rm{d}}}}+{{{{\rm{X}}}}}_{{{{\rm{p}}}}}{}^{{{{\rm{T}}}}}{{{\rm{p}}}}+{{{\rm{e}}}}$$

where ***m*** represents the measurement bias (4 machines × 1), ***s*** represents the sampling bias of TD (3 sites × 1) and ADHD (3 sites × 1), ***d*** represents the disorder factor (ADHD × 1), and ***p*** represents the participant factor (43 participants with repeated measures × 1). We used ridge regression to calculate the parameters. We also assessed measurement bias and sampling bias by excluding or including participants as a random intercept in the model, detailed in the Supplementary Material. The brain structures were normalized for ridge regression. The model was tested on raw CDM data, TS-corrected CDM data, and ComBat-corrected CDM data to analyze measurement and sampling bias before and after harmonization. Measurement bias was calculated as the average of the effect sizes of the brain structures across different MRI scanners. The sampling biases in participants with TD and patients with ADHD were defined separately as the average effect sizes of the brain structures across different sites.

### Statistical analyses

We used R (version 4.3.1; The R Foundation for Statistical Computing, Vienna, Austria) and Python (version 3.11.6; Python Software Foundation, Wilmington, DE, USA) for statistical analyses. First, we examined the necessity and validity of harmonization. We used a repeated-measures analysis of variance (ANOVA) on the TS dataset to examine the necessity of harmonization. Additionally, we computed the intraclass correlation coefficient (ICC) of the harmonized structural brain of the TS dataset, a descriptive statistic that can be used when quantitative measurements are made on units organized into groups (the individuals in this study) to examine validity [22]. We compared the ICC among the raw, TS-corrected, and ComBat-corrected data of the TS dataset using ANOVA, followed by a post hoc test using Tukey’s Honest Significant Difference (HSD) method with family-wise error rate (FWE) correction. We subsequently adapted TS and ComBat to correct the brain structure data in 178 children with TD and 116 children with ADHD from the CDM project. We calculated the measurement and sampling biases for TD and ADHD and compared these biases among TS-corrected, ComBat-corrected, and raw data using ANOVA and a post hoc test using Tukey’s HSD method with FWE correction.

Additionally, we examined the association between brain structures and ADHD in CDM dataset. First, we adapted a linear mixed-effects model to examine the relationship between brain structures harmonized by TS and ADHD. We analyzed this model using the R-package “lmerTest”. For the mixed-effects model with a group (ADHD or TD) as the independent variable and brain structures as the dependent variable, we considered participants’ age, sex, handedness, intelligence quotient (IQ) measured using the Wechsler Intelligence Scale for Children (WISC), and intracranial volume of the brain as covariates. As some participants participated in the experiment multiple times, the subject ID (used to distinguish whether it was the same person) was modeled as a random effect. Raw brain structural data and brain structures harmonized using ComBat were also used in the mixed-effects model to compare children with ADHD and TD. Additionally, considering the differences in age, sex, and handedness between the ADHD and TD groups, we adapted the propensity score matching method to match the age, sex, and handedness of the TD group with the ADHD group (*N* = 94) by using the R package “Matching” with caliper = 0.25, and analyzed them similarly [23, 24]. Specifically, after matching, we conducted mixed-effects regressions to examine the differences between ADHD and TD, with group (ADHD or TD) as the independent variable and brain structures as the dependent variable, controlling for IQ and ICV as covariates. In the current study, for analyses involving brain structures, we applied false discovery rate (FDR) correction to 82 brain regions, 68 cortical regions and 14 subcortical regions, for multiple comparisons correction [25].

## Results

### Necessity of harmonization

Table S3 summarizes the results of the repeated-measures ANOVA of the raw data from the TS dataset. The results showed that 40/82 brain regions showed significant differences among the same participants after false discovery rate (FDR) correction (Table S3 and Fig. S1). However, after harmonization by TS and ComBat methods, there were no differences across four MRI scanners (Tables S4, S5).

### Validity of traveling-subject harmonization

**Fig. 1:**
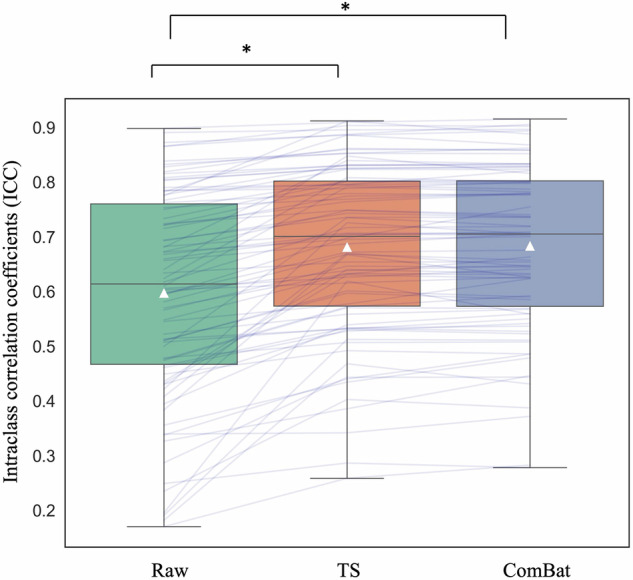
Comparison of the intraclass correlation coefficient (ICC) among TS-corrected, ComBat-corrected, and raw brain structure data in traveling subjects. The ICC of the TS-corrected data (M = 0.681, SD = 0.155) was larger than that of the raw data (M = 0.597, SD = 0.189). There was no significant difference between TS-corrected and ComBat-corrected data (M = 0.683, SD = 0.151) of the traveling subjects. Additionally, the ICC of the ComBat-corrected data was larger than that of the raw data. *p < 0.05.

First, we compared the ICC among TS-corrected, ComBat-corrected, and raw data from 14 traveling subjects. ANOVA showed a significant difference among the data (*F*(2,243) = 7.16, *p* < 0.01) (Fig. 1). The post hoc test showed that the ICC of the TS-corrected data (M = 0.681, SD = 0.155) was larger than that of the raw data (M = 0.597, SD = 0.189) (FWE *p* < 0.05). There was no significant difference between TS-corrected and ComBat-corrected data (M = 0.683, SD = 0.151) of the traveling subjects (FWE *p* = 0.996). Additionally, the ICC of the ComBat-corrected data was larger than that of the raw data (FWE *p* < 0.05).

**Fig. 2:**
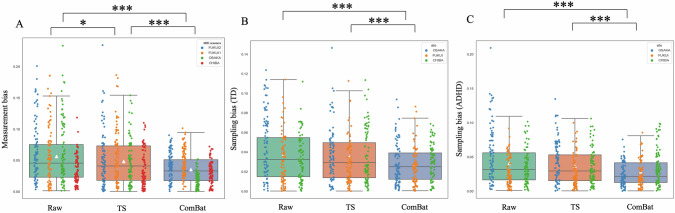
Comparsion of the measurement bias and sampling bias among raw data, TS-corrected data and Combat-corrected data. **A** The measurement bias of TS-corrected data (M = 0.048, SD = 0.022) was smaller than that of the raw data (M = 0.056, SD = 0.024), and larger than that of the ComBat-corrected data (M = 0.031, SD = 0.012). The measurement bias of the ComBat-corrected data was smaller than that of the raw data. **B** The sampling bias in the TS-corrected data (M = 0.035, SD = 0.015) and raw data (M = 0.038, SD = 0.019) of the TD group was greater than that of the ComBat-corrected data (M = 0.025, SD = 0.012). There was no significant difference between TS-corrected and raw data. **C** The sampling bias in the TS-corrected data (M = 0.036, SD = 0.016) and raw data (M = 0.040, SD = 0.018) of the ADHD group was larger than that of the ComBat-corrected data (M = 0.026, SD = 0.011). There was no significant difference between TS-corrected and raw data. ***p < 0.001; *p < 0.05.

Subsequently, we applied TS and ComBat to children from an independent dataset, the CDM project, including children with ADHD and TD, to correct measurement bias due to different MRI machines. We calculated and compared the measurement and sampling biases among TS-corrected, ComBat-corrected, and raw data. ANOVA showed that there were significant differences among the data in terms of measurement bias (*F*(2,243) = 33.492, *p* < 0.001) and sampling bias in the ADHD (*F*(2,243) = 19.856, *p* < 0.001) and TD (*F*(2,243) = 16.792, *p* < 0.001) groups. Subsequently, we conducted a post hoc test to compare the measurement and sampling biases among the different harmonization methods and raw data. The results showed that the measurement bias of TS-corrected data (M = 0.048, SD = 0.022) was smaller than that of the raw data (M = 0.056, SD = 0.024) (FWE *p* = 0.034), while it was larger than that of the ComBat-corrected data (M = 0.031, SD = 0.012) (FWE *p* < 0.001). The measurement bias of the ComBat-corrected data was significantly smaller than that of the raw data (FWE *p* < 0.001) (Fig. 2A). Further, the TS-corrected data (M = 0.035, SD = 0.015) and raw data (M = 0.038, SD = 0.019) had a larger sampling bias than the ComBat-corrected data for the TD group (M = 0.025, SD = 0.012) (TS vs. ComBat: FWE *p* < 0.001; raw vs. ComBat: FWE *p* < 0.001), while no significant difference between TS-corrected data and raw data was observed (FWE *p* = 0.483) (Fig. 2B). The result of sampling bias in the ADHD group was similar to that in the TD group, with the TS-corrected data (M = 0.036, SD = 0.016) and raw data (M = 0.040, SD = 0.018) displaying a larger sampling bias than ComBat-corrected data (M = 0.026, SD = 0.011) (TS vs. ComBat: FWE *p* < 0.001; raw vs. ComBat: FWE *p* < 0.001), while no significant difference between TS-corrected data and raw data was observed (FWE *p* = 0.213) (Fig. 2C). The results of the model with participants as a random intercept are summarized in Fig. S2. The results of the model without the participants factor as a covariate are summarized in Fig. S3, which are similar to the results mentioned in above.

Furthermore, we utilized ANOVA and a post hoc test to compare the raw data, TS-corrected data, and ComBat-corrected data. The results of the ANOVA are summarized in Table S6, while the post hoc results are summarized in Table S7. According to the findings, there was no significant difference between the raw data and ComBat-corrected data. However, 8 brain regions displayed significant differences between the raw data and TS-corrected data. One brain region (left entorhinal cortex) remained significant even after FDR correction. Additionally, the volume of several brain regions (left cuneus, left entorhinal cortex, left pericalcarine cortex, left hippocampus, bilateral amygdala, and right thalamus) showed significant differences between the ComBat-corrected data and TS-corrected data.

### Comparison between ADHD and TD

**Fig. 3:**
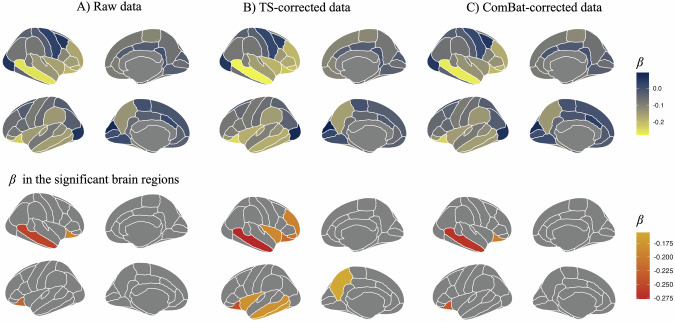
The brain regions with significant differences between the ADHD and TD groups using multiple methods of correction in the mixed-effects model. The continuous color map showed the beta coefficients of each brain region, calculated using mixed effects models. **A** The brain regions, including the right middle temporal gyrus and bilateral orbitofrontal cortex (lateral region), show significant volumetric differences between the ADHD and TD groups in the raw brain structure data. **B** The brain regions, including the bilateral middle temporal gyrus, bilateral orbitofrontal cortex (lateral region), right inferior frontal gyrus (orbital part), right middle frontal gyrus (rostral part), left inferior temporal gyrus, left precuneus cortex, and bilateral insular cortex, show significant volumetric differences between the ADHD and TD groups in TS-corrected brain structure data. **C** The brain regions, including the right middle temporal gyrus and bilateral orbitofrontal cortex (lateral region), show significant volumetric differences between the ADHD and TD groups in the ComBat-corrected brain structure data.

We compared brain structures between the ADHD and TD groups. The results of the raw, TS-corrected, and ComBat-corrected data are summarized in Tables S8, 9, 10, 11. The standardized coefficients of each type of data in all brain regions and significant brain regions are summarized in Fig. 3. In the raw data, the ADHD group had significantly smaller brain volumes than the TD group in the right middle temporal gyrus (*β* = −0.255, 95% Confidence Interval (CI) = −0.366 to −0.145, FDR *p* = 0.001) and bilateral orbitofrontal cortex (lateral region) (right: *β* = −0.193, 95% CI = −0.296 to −0.090, FDR *p* = 0.008; left: *β* = −0.226, 95% CI = −0.326 to −0.126, FDR *p* = 0.001) (Fig. 3A), whereas in the TS-corrected data, the brain volume in the bilateral middle temporal gyrus (left: *β* = −0.179, 95% CI = −0.292 to −0.065, FDR *p* = 0.031; right: *β* = −0.277, 95% CI = −0.391 to −0.162, FDR *p* < 0.001), bilateral orbitofrontal cortex (lateral region) (right: *β* = −0.219, 95% CI = −0.325 to −0.113, FDR *p* = 0.002; left: *β* = −0.246, 95% CI = −0.353 to −0.139, FDR *p* < 0.001), right inferior frontal gyrus (orbital part) (*β* = −0.209, 95% CI = −0.353 to −0.139, FDR *p* = 0.027), right middle frontal gyrus (rostral part) (*β* = −0.195, 95% CI = −0.319 to −0.072, FDR *p* = 0.031), left inferior temporal gyrus (*β* = −0.169, 95% CI = −0.282 to −0.057, FDR *p* = 0.037), left precuneus cortex (*β* = −0.155, 95% CI = −0.261 to −0.049, FDR *p* = 0.037), and bilateral insular cortex (left: *β* = −0.188, 95% CI = −0.303 to −0.057, FDR *p* = 0.037; right: *β* = −0.180, 95% CI = −0.316 to −0.060, FDR *p* = 0.037) were significantly smaller than that in the TD group (Fig. 3B). Regarding ComBat-corrected data, the right middle temporal gyrus (*β* = −0.267, 95% CI = −0.382 to −0.152, FDR *p* = 0.001) and bilateral orbitofrontal cortex (lateral region) (right: *β* = −0.199, 95% CI = −0.307 to −0.091, FDR *p* = 0.010; left: *β* = −0.235, 95% CI = −0.341 to −0.130, FDR *p* = 0.001) showed significant differences between the ADHD and TD groups (Fig. 3C).

To obtain more robust results, we matched the participants by propensity score, including age, sex, and handedness, and the data of 188 participants (94 ADHD and 94 TD) were included in the analysis. The results are summarized in the Supplementary Material. The result showed that only the right middle temporal gyrus (TS: *β* = −0.265, 95% CI = −0.413 to −0.117, FDR *p* = 0.047; ComBat: *β* = −0.271, 95% CI = −0.408 to −0.134, FDR *p* = 0.014) was smaller in children with ADHD compared with the children with TD in TS-corrected and ComBat-corrected data. For raw data, the right middle temporal gyrus (*β* = −0.283, 95% CI = −0.421 to −0.146, FDR *p* = 0.007) and left orbitofrontal cortex (*β* = −0.215, 95% CI = −0.334 to −0.096, FDR *p* = 0.020) showed significant differences between the ADHD and TD groups. The results are summarized in Table S12.

## Discussion

This study aimed to examine the structural brain differences between children with ADHD and children with TD by addressing the measurement bias in MRI data from different scanners using the TS method. This study demonstrates that the TS harmonization method can improve the reliability of structural brain data across multiple MRI scanners by applying the TS method to an independent dataset. This study also examined the differences between ADHD and TD to elucidate the brain structural characteristics of ADHD by comparing different harmonization methods and raw data. The results of TS-corrected data showed volumetric differences between the ADHD and TD groups in the cortical regions, including the bilateral middle temporal and bilateral orbitofrontal cortex, right inferior frontal gyrus, right middle frontal gyrus, left inferior temporal gyrus, left precuneus cortex, and bilateral insular cortex. In contrast, comparisons between ADHD and TD using ComBat-corrected and raw data showed different findings than when using TS-corrected data.

Before harmonization, the current study provided evidence that half of the brain regions showed significant differences among the same participants, demonstrating the necessity of harmonization for measurement bias in MRI scanners. The TS method, which estimates measurement bias more accurately [17], significantly reduces measurement bias compared with the raw data. This method involves considerable logistical effort, including TS recruitment and scan scheduling within a short duration; however, it corrects for measurement bias. The TS method addresses the variations introduced by different scanners and scanning protocols [17]. This is crucial for multi-site studies, wherein such variations can compromise the credibility of research findings. Although a previous study proved the validity of TS in structural brain data [18], few studies have applied TS to correct for measurement bias in an independent dataset. One of the contributions of this study is the validation of the TS method in structural brain data from an independent dataset, providing evidence that this method can be effectively used to harmonize multi-site MRI bias in structural brain data.

This study underscores the importance of harmonization methods in multi-site neuroimaging studies. Site differences consist of two types of bias: measurement and sampling. Measurement bias includes differences in the properties of MRI scanners, such as imaging variables, field strength, MRI manufacturers, and scanner models, whereas sampling bias refers to differences in participant groups between sites. The measurement bias can influence the accuracy of results in multi-site studies. Sampling bias can be considered as the biological part of site difference because of sampling from different subpopulations [17], which makes it possible to include the biological part of disorders. Therefore it is inevitable that the data will contain sampling bias. Since the sampling bias included the biological part of the site difference, the reduction of sampling bias may influence the analysis of the difference in brain structures between ADHD and TD groups. The current study calculated the sampling bias is consistent to the previous study, which proved the existence of sampling bias in our dataset by calculating the sampling bias from the raw data, and ComBat method could reduce it as measurement bias. Although ComBat can provide more power to reduce the measurement bias, the overcorrection of sampling bias may influence the accuracy of findings.

The results indicated that TS-corrected data exhibited a smaller measurement bias compared with the raw data, but a larger measurement bias than ComBat-corrected data. This suggests that while the TS method is effective in reducing some measurement biases inherent in multi-site data collection, the ComBat method achieves a greater reduction in measurement bias. This result is consistent with that of a previous study that proved the effectiveness of robust normalization methods for MRI data [16]. Our analysis also showed that ComBat-corrected data had a smaller sampling bias than the raw and TS-corrected data.

These results emphasize the importance of selecting appropriate correction methods for multi-site studies. While TS methods offer improvements over raw data and do not influence the sampling bias of different sites, the ComBat method’s rigorous statistical framework provides superior performance in reducing measurement bias while also reducing sampling biases as measurement bias. Considering that reducing the sampling bias in patients influences the findings of the neural basis of a disorder [17] and that sampling bias does exist in raw data, harmonization using the TS method may be more appropriate for the current analysis.

Analysis using TS-corrected data revealed significant volumetric differences between the ADHD and TD groups in several brain regions. Notably, patients with ADHD exhibited smaller volumes in the bilateral middle temporal gyrus, bilateral orbitofrontal cortex, right inferior frontal gyrus, right middle frontal gyrus, left inferior temporal gyrus, left precuneus, and bilateral insular cortex. Reductions in the bilateral middle temporal gyrus and orbitofrontal cortex have been consistently reported [8, 12, 26, 27]. These regions are crucial for cognitive functions such as information processing, attention regulation, and emotional control, which are often impaired in ADHD [28, 29]. Moreover, Castellanos and Aoki’s [30] suggestion that ADHD is a disorder of the default mode network (DMN) and Tamm et al.’s. [31] findings of frontotemporal dysfunction align with the current study’s results, particularly the consistent finding of abnormalities in the temporal gyrus. The middle temporal gyrus is involved in DMN [32], and ADHD showed decreased functional connectivity of the middle temporal gyrus as part of the DMN with other networks [33, 34]. In the current study, even when comparing the ADHD group to a matched TD group, differences in the middle temporal gyrus remained significant, emphasizing the robustness of these findings and suggesting its potential use as a biomarker for ADHD. The TS method can reduce the measurement bias derived from multiple MRI scanners, and the results of the raw data may be influenced by this bias. Additionally, as the comparison of TS-corrected data with ComBat-corrected data revealed some differences in results, we considered that the excessive reduction of sampling bias when using ComBat may influence the accuracy of findings. Previous studies that did not use the TS method could not accurately correct these biases, which may have led to the discrepancies in the results.

In addition to the brain regions mentioned above, the right inferior frontal gyrus and the right middle frontal gyrus also show reduced volumes in patients with ADHD. These frontal regions are associated with executive functions, including inhibitory control and working memory, and their reduced volume may contribute to the characteristic impulsivity and inattention observed in ADHD [35, 36]. Furthermore, the current study has reported volume reductions in the left inferior temporal gyrus and left precuneus cortex in individuals with ADHD. The inferior temporal gyrus is involved in visual processing and object recognition [37], whereas the precuneus is involved in self-referential thinking and visuospatial processing [38]. These structural differences may underlie some cognitive and perceptual challenges faced by patients with ADHD. Finally, the bilateral insular cortex, which is involved in perceptual awareness and emotional regulation [39], has also been shown to have a reduced volume in individuals with ADHD. The role of the insula in integrating emotional and cognitive processes suggests that its structural abnormalities could be linked to the emotional dysregulation frequently observed in ADHD [40, 41]. Overall, these findings highlight a widespread pattern of cortical volume reduction in ADHD, which encompasses the regions involved in attention, executive function, emotional regulation, and cognitive processing. These structural abnormalities underscore the complexity of ADHD and the need for a comprehensive approach to its study and treatment. The observed structural differences in these brain regions align with previous neuroimaging studies, implicating alterations in the neural circuitry associated with attention, executive function, emotional regulation, and cognitive control in individuals with ADHD. However, these regions showed no significant differences between the ADHD and TD groups after comparing the ADHD group with a matched TD group, implying that these regions may be influenced by age, sex, and handedness.

Despite these promising results, this study had some limitations. The study sample may not fully represent the broader population of children with ADHD. The participants were drawn from specific geographical regions and clinical settings, which could limit the generalizability of the findings to other populations. Additionally, this study only examined the brain structure characteristics in children with ADHD elucidated using harmonization. For functional brain, Castellanos et al. [30] reported that children with ADHD have abnormalities in the default mode network and network of brain regions involved in the reward system, while a recent meta-analysis study of resting-state functional MRI reported that no findings specific to ADHD could be obtained [42]. Future studies should consider examining the functional brain characteristics in children with ADHD by using the TS method.

In conclusion, this study demonstrated the effectiveness of the TS method in correcting measurement bias in multi-site MRI studies involving children with ADHD. These findings highlight significant structural differences in the brains of patients with ADHD, particularly in the middle temporal gyrus, and underscore the importance of using robust harmonization techniques to improve the reproducibility and accuracy of neuroimaging research.

## Supplementary information

Supplementary Material

## Data Availability

The code used to analyze and create the manuscript is available at https://osf.io/6ay5q/?view_only=30bc49dbce1f490c9b4dc4e7cdb0bc85.
