# Supplementary material for: Brain structure characteristics in children with attention-deficit/hyperactivity disorder elucidated using traveling-subject harmonization
Source: Mol Psychiatry. 2025 Aug 8;30(12):5873–9. doi: 10.1038/s41380-025-03142-6 (PMC12602332; doi:10.1038/s41380-025-03142-6)
Supplement: Supplementary file 1 — Supplementary Material [file 41380_2025_3142_MOESM1_ESM.docx]

**Supplementary Material**

**Brain structure characteristics in children with attention-deficit/hyperactivity disorder elucidated using travelling-subjects harmonization.**

**Subjects and Methods**

**Measurement and sampling biases of different harmonization methods**

Measurement and sampling biases of different harmonization methods were assessed in the study.

The model incorporating participants as the random intercept was as follows:

Brain structures = x_m_^T^m + x_s_^T^s + x_d_^T^d + x_p_^T^p + e,

where ***m*** represents the measurement bias (4 machines × 1), ***s*** represents the sampling bias of TD (3 sites × 1) and ADHD (3 sites × 1), and ***d*** represents the disorder factor (ADHD × 1).

The model without participants as a covariate was:

Brain structures = x_m_^T^m + x_s_^T^s + x_d_^T^d + e,

where ***m*** represents the measurement bias (4 machines × 1), ***s*** represents the sampling bias of TD (3 sites × 1) and ADHD (3 sites × 1), and ***d*** represents the disorder factor (ADHD × 1).

**Table S1 The demographic data of each scanner**

|  | Uni. of Fukui Ⅰ | | Uni. of Fukui Ⅱ | | Osaka Uni. | | Chiba Uni. | |
| --- | --- | --- | --- | --- | --- | --- | --- | --- |
|  | TD(n=37) | ADHD(n=19) | TD(n=91) | ADHD(n=82) | TD(n=9) | ADHD(n=19) | TD(n=45) | ADHD(n=1) |
| Age (years) | 13.22±2.99 | 9.86±1.53 | 12.18±2.64 | 10.53±2.16 | 8.83±2.14 | 9.32±2.88 | 14.33±2.23 | 11.83 |
| Sex |  |  |  |  |  |  |  |  |
| Male/Female(n) | 19/18 | 15/4 | 69/18 | 74/3 | 5/4 | 18/1 | 26/19 | 1/0 |
| Handedness(n) |  |  |  |  |  |  |  |  |
| Right/Left/Ambidextrous (n) | 36/1/0 | 16/3/0 | 86/4/1 | 71/10/1 | 8/1/0 | 18/1/0 | 37/6/2 | 1/0/0 |
| IQ | 106.19±11.69 | 99.47 ±11.43 | 105.13±10.47 | 95.71±13.16 | 91.33±12.85 | 88.12±12.83 | 107.10±12.72 | 81.00 |
| ICV (cm^3^) | 1525.79±173.36 | 1512.97±172.53 | 1373.48±153.62 | 1392.60±162.02 | 1216.84±108.12 | 1228.49±136.26 | 1359.40±167.10 | 1341.33 |

**Table S2 Scanning parameters for each scanner**

| MRI scanner | Head coil | Scan time (min: sec) | TR (ms) | TE (ms) | FA (deg) | FOV (mm) | Matrix | Voxel dimension (mm) | Slice thickness | Slice |
| --- | --- | --- | --- | --- | --- | --- | --- | --- | --- | --- |
| University of Fukui (PET/MR) | 8 ch | 04:54 | 8.5 | 3.2 | 11 | 256*256 | 256*256 | 1*1*1 | 1 (gap 0) | 176 |
| University of Fukui (MR750) | 32 ch | 05:00 | 6.4 | 2 | 11 | 256*256 | 256*256 | 1*1*1 | 1 (gap 0) | 172 |
| Osaka University (Architect) | 48 ch | 05:10 | 876.332 | 0.02 | 5 | 240*240 | 240*240 | 0.938*0.938*0.5 | 1 | 480 |
| Chiba University | 32 ch | 04:31 | 8.124 | 3.164 | 15 | 256*256 | 256*256 | 1*1*1 | 1 (gap 0) | 178 |

min = minute

sec = second

TR = repetition time

TE = echo time

FA = flip angle

FOV = field of view

ch = channels

**Table S3 The results of repeated measures ANOVA for raw data**

| ROI | *F* | *p* | FDR *p* |
| --- | --- | --- | --- |
| Left-Pallidum | 36.451 | <0.001 | <0.001 |
| Right-Thalamus | 25.483 | <0.001 | <0.001 |
| Left-Thalamus | 24.232 | <0.001 | <0.001 |
| Left-Caudate | 24.212 | <0.001 | <0.001 |
| lh_parahippocampal_volume | 21.797 | <0.001 | <0.001 |
| Right-Pallidum | 20.622 | <0.001 | <0.001 |
| Left-Amygdala | 18.574 | <0.001 | <0.001 |
| rh_medialorbitofrontal_volume | 18.482 | <0.001 | <0.001 |
| lh_entorhinal_volume | 18.112 | <0.001 | <0.001 |
| Left-Hippocampus | 17.413 | <0.001 | <0.001 |
| rh_inferiorparietal_volume | 16.886 | <0.001 | <0.001 |
| Right-Hippocampus | 15.959 | <0.001 | <0.001 |
| Right-Caudate | 14.105 | <0.001 | <0.001 |
| lh_medialorbitofrontal_volume | 12.817 | <0.001 | <0.001 |
| Right-Amygdala | 12.217 | <0.001 | <0.001 |
| rh_parahippocampal_volume | 8.565 | <0.001 | 0.001 |
| Right-Accumbens-area | 7.842 | <0.001 | 0.001 |
| rh_frontalpole_volume | 7.467 | <0.001 | 0.002 |
| rh_parsorbitalis_volume | 6.424 | 0.001 | 0.005 |
| lh_parsorbitalis_volume | 6.416 | 0.001 | 0.005 |
| lh_superiortemporal_volume | 5.765 | 0.002 | 0.009 |
| rh_superiortemporal_volume | 5.744 | 0.002 | 0.009 |
| lh_lateralorbitofrontal_volume | 5.510 | 0.003 | 0.011 |
| lh_cuneus_volume | 5.162 | 0.004 | 0.015 |
| lh_caudalmiddlefrontal_volume | 5.052 | 0.005 | 0.016 |
| lh_parsopercularis_volume | 5.038 | 0.005 | 0.016 |
| lh_frontalpole_volume | 4.963 | 0.005 | 0.016 |
| rh_entorhinal_volume | 4.825 | 0.006 | 0.017 |
| rh_superiorparietal_volume | 4.816 | 0.006 | 0.017 |
| rh_bankssts_volume | 4.803 | 0.006 | 0.017 |
| Left-Putamen | 4.734 | 0.007 | 0.018 |
| rh_cuneus_volume | 4.690 | 0.007 | 0.018 |
| rh_inferiortemporal_volume | 4.643 | 0.007 | 0.018 |
| rh_temporalpole_volume | 4.483 | 0.008 | 0.021 |
| lh_pericalcarine_volume | 4.380 | 0.009 | 0.022 |
| lh_rostralanteriorcingulate_volume | 4.365 | 0.010 | 0.022 |
| rh_parsopercularis_volume | 4.339 | 0.010 | 0.022 |
| lh_fusiform_volume | 4.334 | 0.010 | 0.022 |
| lh_inferiorparietal_volume | 4.164 | 0.012 | 0.025 |
| Left-Accumbens-area | 4.103 | 0.013 | 0.026 |
| Right-Putamen | 3.495 | 0.024 | 0.049 |
| lh_bankssts_volume | 3.387 | 0.027 | 0.054 |
| lh_lingual_volume | 3.321 | 0.030 | 0.057 |
| lh_superiorparietal_volume | 3.305 | 0.030 | 0.057 |
| rh_parstriangularis_volume | 3.216 | 0.033 | 0.061 |
| rh_lateralorbitofrontal_volume | 3.074 | 0.039 | 0.070 |
| rh_supramarginal_volume | 2.997 | 0.042 | 0.074 |
| rh_middletemporal_volume | 2.769 | 0.054 | 0.094 |
| lh_lateraloccipital_volume | 2.637 | 0.063 | 0.107 |
| lh_caudalanteriorcingulate_volume | 2.564 | 0.069 | 0.113 |
| rh_rostralmiddlefrontal_volume | 2.528 | 0.071 | 0.116 |
| lh_insula_volume | 2.189 | 0.105 | 0.166 |
| lh_precuneus_volume | 2.086 | 0.118 | 0.183 |
| lh_rostralmiddlefrontal_volume | 2.068 | 0.120 | 0.184 |
| rh_rostralanteriorcingulate_volume | 2.047 | 0.123 | 0.185 |
| lh_parstriangularis_volume | 1.983 | 0.132 | 0.195 |
| rh_lingual_volume | 1.864 | 0.152 | 0.219 |
| rh_isthmuscingulate_volume | 1.736 | 0.176 | 0.249 |
| lh_supramarginal_volume | 1.706 | 0.182 | 0.250 |
| rh_precuneus_volume | 1.702 | 0.182 | 0.250 |
| lh_inferiortemporal_volume | 1.678 | 0.188 | 0.253 |
| rh_superiorfrontal_volume | 1.664 | 0.190 | 0.253 |
| rh_transversetemporal_volume | 1.618 | 0.201 | 0.262 |
| lh_temporalpole_volume | 1.560 | 0.215 | 0.276 |
| lh_precentral_volume | 1.484 | 0.234 | 0.296 |
| lh_postcentral_volume | 1.416 | 0.253 | 0.310 |
| lh_superiorfrontal_volume | 1.415 | 0.253 | 0.310 |
| lh_posteriorcingulate_volume | 1.321 | 0.282 | 0.340 |
| lh_middletemporal_volume | 1.294 | 0.290 | 0.346 |
| rh_caudalmiddlefrontal_volume | 1.214 | 0.317 | 0.373 |
| lh_transversetemporal_volume | 1.196 | 0.324 | 0.375 |
| rh_pericalcarine_volume | 1.118 | 0.354 | 0.403 |
| rh_posteriorcingulate_volume | 1.051 | 0.381 | 0.425 |
| rh_insula_volume | 1.047 | 0.383 | 0.425 |
| rh_lateraloccipital_volume | 1.023 | 0.393 | 0.430 |
| rh_caudalanteriorcingulate_volume | 0.853 | 0.473 | 0.511 |
| lh_paracentral_volume | 0.800 | 0.502 | 0.535 |
| lh_isthmuscingulate_volume | 0.670 | 0.575 | 0.605 |
| rh_precentral_volume | 0.611 | 0.612 | 0.635 |
| rh_paracentral_volume | 0.393 | 0.758 | 0.778 |
| rh_fusiform_volume | 0.265 | 0.850 | 0.861 |
| rh_postcentral_volume | 0.087 | 0.967 | 0.967 |

ROI = Regions of interest

FDR = false discovery rate

**Table S4 The results of repeated measures ANOVA for TS-corrected data**

| ROI | *F* | *p* | FDR *p* |
| --- | --- | --- | --- |
| lh_bankssts_volume | 1.73E-06 | 1.000 | 1.000 |
| lh_caudalanteriorcingulate_volume | 1.31E-06 | 1.000 | 1.000 |
| lh_caudalmiddlefrontal_volume | 2.57E-06 | 1.000 | 1.000 |
| lh_cuneus_volume | 2.63E-06 | 1.000 | 1.000 |
| lh_entorhinal_volume | 9.23E-06 | 1.000 | 1.000 |
| lh_fusiform_volume | 2.21E-06 | 1.000 | 1.000 |
| lh_inferiorparietal_volume | 2.12E-06 | 1.000 | 1.000 |
| lh_inferiortemporal_volume | 8.55E-07 | 1.000 | 1.000 |
| lh_isthmuscingulate_volume | 3.42E-07 | 1.000 | 1.000 |
| lh_lateraloccipital_volume | 1.34E-06 | 1.000 | 1.000 |
| lh_lateralorbitofrontal_volume | 2.81E-06 | 1.000 | 1.000 |
| lh_lingual_volume | 1.69E-06 | 1.000 | 1.000 |
| lh_medialorbitofrontal_volume | 6.53E-06 | 1.000 | 1.000 |
| lh_middletemporal_volume | 6.59E-07 | 1.000 | 1.000 |
| lh_parahippocampal_volume | 1.11E-05 | 1.000 | 1.000 |
| lh_paracentral_volume | 4.07E-07 | 1.000 | 1.000 |
| lh_parsopercularis_volume | 2.57E-06 | 1.000 | 1.000 |
| lh_parsorbitalis_volume | 3.27E-06 | 1.000 | 1.000 |
| lh_parstriangularis_volume | 1.01E-06 | 1.000 | 1.000 |
| lh_pericalcarine_volume | 2.23E-06 | 1.000 | 1.000 |
| lh_postcentral_volume | 7.22E-07 | 1.000 | 1.000 |
| lh_posteriorcingulate_volume | 6.73E-07 | 1.000 | 1.000 |
| lh_precentral_volume | 7.56E-07 | 1.000 | 1.000 |
| lh_precuneus_volume | 1.06E-06 | 1.000 | 1.000 |
| lh_rostralanteriorcingulate_volume | 2.22E-06 | 1.000 | 1.000 |
| lh_rostralmiddlefrontal_volume | 1.05E-06 | 1.000 | 1.000 |
| lh_superiorfrontal_volume | 7.21E-07 | 1.000 | 1.000 |
| lh_superiorparietal_volume | 1.68E-06 | 1.000 | 1.000 |
| lh_superiortemporal_volume | 2.94E-06 | 1.000 | 1.000 |
| lh_supramarginal_volume | 8.69E-07 | 1.000 | 1.000 |
| lh_frontalpole_volume | 2.53E-06 | 1.000 | 1.000 |
| lh_temporalpole_volume | 7.95E-07 | 1.000 | 1.000 |
| lh_transversetemporal_volume | 6.09E-07 | 1.000 | 1.000 |
| lh_insula_volume | 1.12E-06 | 1.000 | 1.000 |
| rh_bankssts_volume | 2.45E-06 | 1.000 | 1.000 |
| rh_caudalanteriorcingulate_volume | 4.35E-07 | 1.000 | 1.000 |
| rh_caudalmiddlefrontal_volume | 6.19E-07 | 1.000 | 1.000 |
| rh_cuneus_volume | 2.39E-06 | 1.000 | 1.000 |
| rh_entorhinal_volume | 2.46E-06 | 1.000 | 1.000 |
| rh_fusiform_volume | 1.35E-07 | 1.000 | 1.000 |
| rh_inferiorparietal_volume | 8.60E-06 | 1.000 | 1.000 |
| rh_inferiortemporal_volume | 2.37E-06 | 1.000 | 1.000 |
| rh_isthmuscingulate_volume | 8.84E-07 | 1.000 | 1.000 |
| rh_lateraloccipital_volume | 5.21E-07 | 1.000 | 1.000 |
| rh_lateralorbitofrontal_volume | 1.57E-06 | 1.000 | 1.000 |
| rh_lingual_volume | 9.50E-07 | 1.000 | 1.000 |
| rh_medialorbitofrontal_volume | 9.42E-06 | 1.000 | 1.000 |
| rh_middletemporal_volume | 1.41E-06 | 1.000 | 1.000 |
| rh_parahippocampal_volume | 4.36E-06 | 1.000 | 1.000 |
| rh_paracentral_volume | 2.00E-07 | 1.000 | 1.000 |
| rh_parsopercularis_volume | 2.21E-06 | 1.000 | 1.000 |
| rh_parsorbitalis_volume | 3.27E-06 | 1.000 | 1.000 |
| rh_parstriangularis_volume | 1.64E-06 | 1.000 | 1.000 |
| rh_pericalcarine_volume | 5.70E-07 | 1.000 | 1.000 |
| rh_postcentral_volume | 4.41E-08 | 1.000 | 1.000 |
| rh_posteriorcingulate_volume | 5.35E-07 | 1.000 | 1.000 |
| rh_precentral_volume | 3.12E-07 | 1.000 | 1.000 |
| rh_precuneus_volume | 8.67E-07 | 1.000 | 1.000 |
| rh_rostralanteriorcingulate_volume | 1.04E-06 | 1.000 | 1.000 |
| rh_rostralmiddlefrontal_volume | 1.29E-06 | 1.000 | 1.000 |
| rh_superiorfrontal_volume | 8.48E-07 | 1.000 | 1.000 |
| rh_superiorparietal_volume | 2.45E-06 | 1.000 | 1.000 |
| rh_superiortemporal_volume | 2.93E-06 | 1.000 | 1.000 |
| rh_supramarginal_volume | 1.53E-06 | 1.000 | 1.000 |
| rh_frontalpole_volume | 3.80E-06 | 1.000 | 1.000 |
| rh_temporalpole_volume | 2.28E-06 | 1.000 | 1.000 |
| rh_transversetemporal_volume | 8.24E-07 | 1.000 | 1.000 |
| rh_insula_volume | 5.33E-07 | 1.000 | 1.000 |
| Left-Thalamus | 1.23E-05 | 1.000 | 1.000 |
| Left-Caudate | 1.23E-05 | 1.000 | 1.000 |
| Left-Putamen | 2.41E-06 | 1.000 | 1.000 |
| Left-Pallidum | 1.86E-05 | 1.000 | 1.000 |
| Left-Hippocampus | 8.87E-06 | 1.000 | 1.000 |
| Left-Amygdala | 9.46E-06 | 1.000 | 1.000 |
| Left-Accumbens-area | 2.09E-06 | 1.000 | 1.000 |
| Right-Thalamus | 1.30E-05 | 1.000 | 1.000 |
| Right-Caudate | 7.19E-06 | 1.000 | 1.000 |
| Right-Putamen | 1.78E-06 | 1.000 | 1.000 |
| Right-Pallidum | 1.05E-05 | 1.000 | 1.000 |
| Right-Hippocampus | 8.13E-06 | 1.000 | 1.000 |
| Right-Amygdala | 6.22E-06 | 1.000 | 1.000 |
| Right-Accumbens-area | 4.00E-06 | 1.000 | 1.000 |

ROI = Regions of interest

FDR = false discovery rate

**Table S5 The results of repeated measures ANOVA for ComBat-corrected data**

| ROI | | *F* | | *p* | | FDR *p* | |
| --- | --- | --- | --- | --- | --- | --- | --- |
| lh_bankssts_volume | 0.419 | | 0.741 | | 0.999 | |  |
| lh_caudalanteriorcingulate_volume | 0.404 | | 0.751 | | 0.999 | |  |
| lh_caudalmiddlefrontal_volume | 0.539 | | 0.659 | | 0.999 | |  |
| lh_cuneus_volume | 0.955 | | 0.423 | | 0.999 | |  |
| lh_entorhinal_volume | 1.417 | | 0.252 | | 0.999 | |  |
| lh_fusiform_volume | 0.263 | | 0.851 | | 0.999 | |  |
| lh_inferiorparietal_volume | 0.169 | | 0.917 | | 0.999 | |  |
| lh_inferiortemporal_volume | 0.217 | | 0.884 | | 0.999 | |  |
| lh_isthmuscingulate_volume | 0.140 | | 0.936 | | 0.999 | |  |
| lh_lateraloccipital_volume | 0.321 | | 0.810 | | 0.999 | |  |
| lh_lateralorbitofrontal_volume | 0.208 | | 0.891 | | 0.999 | |  |
| lh_lingual_volume | 0.492 | | 0.690 | | 0.999 | |  |
| lh_medialorbitofrontal_volume | 0.777 | | 0.514 | | 0.999 | |  |
| lh_middletemporal_volume | 0.185 | | 0.906 | | 0.999 | |  |
| lh_parahippocampal_volume | 1.933 | | 0.140 | | 0.999 | |  |
| lh_paracentral_volume | 0.150 | | 0.929 | | 0.999 | |  |
| lh_parsopercularis_volume | 1.037 | | 0.387 | | 0.999 | |  |
| lh_parsorbitalis_volume | 0.536 | | 0.660 | | 0.999 | |  |
| lh_parstriangularis_volume | 0.270 | | 0.847 | | 0.999 | |  |
| lh_pericalcarine_volume | 0.835 | | 0.483 | | 0.999 | |  |
| lh_postcentral_volume | 0.507 | | 0.679 | | 0.999 | |  |
| lh_posteriorcingulate_volume | 0.187 | | 0.904 | | 0.999 | |  |
| lh_precentral_volume | 0.404 | | 0.751 | | 0.999 | |  |
| lh_precuneus_volume | 0.438 | | 0.727 | | 0.999 | |  |
| lh_rostralanteriorcingulate_volume | 0.385 | | 0.764 | | 0.999 | |  |
| lh_rostralmiddlefrontal_volume | 0.513 | | 0.676 | | 0.999 | |  |
| lh_superiorfrontal_volume | 0.187 | | 0.905 | | 0.999 | |  |
| lh_superiorparietal_volume | 0.625 | | 0.603 | | 0.999 | |  |
| lh_superiortemporal_volume | 0.766 | | 0.520 | | 0.999 | |  |
| lh_supramarginal_volume | 0.264 | | 0.851 | | 0.999 | |  |
| lh_frontalpole_volume | 0.850 | | 0.475 | | 0.999 | |  |
| lh_temporalpole_volume | 0.197 | | 0.897 | | 0.999 | |  |
| lh_transversetemporal_volume | 0.279 | | 0.841 | | 0.999 | |  |
| lh_insula_volume | 0.365 | | 0.779 | | 0.999 | |  |
| rh_bankssts_volume | 0.817 | | 0.492 | | 0.999 | |  |
| rh_caudalanteriorcingulate_volume | 0.046 | | 0.987 | | 0.999 | |  |
| rh_caudalmiddlefrontal_volume | 0.352 | | 0.788 | | 0.999 | |  |
| rh_cuneus_volume | 0.741 | | 0.534 | | 0.999 | |  |
| rh_entorhinal_volume | 0.477 | | 0.700 | | 0.999 | |  |
| rh_fusiform_volume | 0.042 | | 0.989 | | 0.999 | |  |
| rh_inferiorparietal_volume | 1.314 | | 0.284 | | 0.999 | |  |
| rh_inferiortemporal_volume | 0.812 | | 0.495 | | 0.999 | |  |
| rh_isthmuscingulate_volume | 0.366 | | 0.778 | | 0.999 | |  |
| rh_lateraloccipital_volume | 0.185 | | 0.906 | | 0.999 | |  |
| rh_lateralorbitofrontal_volume | 0.190 | | 0.903 | | 0.999 | |  |
| rh_lingual_volume | 0.318 | | 0.812 | | 0.999 | |  |
| rh_medialorbitofrontal_volume | 1.451 | | 0.243 | | 0.999 | |  |
| rh_middletemporal_volume | 0.629 | | 0.601 | | 0.999 | |  |
| rh_parahippocampal_volume | 0.855 | | 0.472 | | 0.999 | |  |
| rh_paracentral_volume | 0.083 | | 0.969 | | 0.999 | |  |
| rh_parsopercularis_volume | 0.537 | | 0.660 | | 0.999 | |  |
| rh_parsorbitalis_volume | 0.678 | | 0.571 | | 0.999 | |  |
| rh_parstriangularis_volume | 0.448 | | 0.720 | | 0.999 | |  |
| rh_pericalcarine_volume | 0.216 | | 0.885 | | 0.999 | |  |
| rh_postcentral_volume | 0.009 | | 0.999 | | 0.999 | |  |
| rh_posteriorcingulate_volume | 0.365 | | 0.779 | | 0.999 | |  |
| rh_precentral_volume | 0.081 | | 0.970 | | 0.999 | |  |
| rh_precuneus_volume | 0.446 | | 0.721 | | 0.999 | |  |
| rh_rostralanteriorcingulate_volume | 0.049 | | 0.985 | | 0.999 | |  |
| rh_rostralmiddlefrontal_volume | 0.533 | | 0.662 | | 0.999 | |  |
| rh_superiorfrontal_volume | 0.350 | | 0.789 | | 0.999 | |  |
| rh_superiorparietal_volume | 0.623 | | 0.604 | | 0.999 | |  |
| rh_superiortemporal_volume | 0.755 | | 0.526 | | 0.999 | |  |
| rh_supramarginal_volume | 0.349 | | 0.790 | | 0.999 | |  |
| rh_frontalpole_volume | 0.660 | | 0.582 | | 0.999 | |  |
| rh_temporalpole_volume | 0.348 | | 0.791 | | 0.999 | |  |
| rh_transversetemporal_volume | 0.358 | | 0.784 | | 0.999 | |  |
| rh_insula_volume | 0.257 | | 0.856 | | 0.999 | |  |
| Left-Thalamus | 3.599 | | 0.022 | | 0.617 | |  |
| Left-Caudate | 4.632 | | 0.007 | | 0.596 | |  |
| Left-Putamen | 0.654 | | 0.585 | | 0.999 | |  |
| Left-Pallidum | 3.566 | | 0.023 | | 0.617 | |  |
| Left-Hippocampus | 1.289 | | 0.292 | | 0.999 | |  |
| Left-Amygdala | 1.660 | | 0.191 | | 0.999 | |  |
| Left-Accumbens-area | 0.664 | | 0.579 | | 0.999 | |  |
| Right-Thalamus | 2.484 | | 0.075 | | 0.999 | |  |
| Right-Caudate | 3.009 | | 0.042 | | 0.854 | |  |
| Right-Putamen | 0.637 | | 0.595 | | 0.999 | |  |
| Right-Pallidum | 2.629 | | 0.064 | | 0.999 | |  |
| Right-Hippocampus | 1.054 | | 0.380 | | 0.999 | |  |
| Right-Amygdala | 0.994 | | 0.406 | | 0.999 | |  |
| Right-Accumbens-area | 0.591 | | 0.625 | | 0.999 | |  |

ROI = Regions of interest

FDR = false discovery rate

| **Table S6**  **The result of ANOVA among the volumes among raw data, TS-corrected data and ComBat-corrected data** | | | | | | |
| --- | --- | --- | --- | --- | --- | --- |
| ROI | Sum of Squared | df1 | df2 | F | p | FDR p |
| lh_bankssts_volume | 301922.09 | 2 | 243 | 0.397 | 0.673 | 0.956 |
| lh_caudalanteriorcingulate_volume | 77203.18 | 2 | 243 | 0.140 | 0.870 | 0.986 |
| lh_caudalmiddlefrontal_volume | 1980006.97 | 2 | 243 | 0.373 | 0.689 | 0.956 |
| lh_cuneus_volume | 5727050.21 | 2 | 243 | 6.016 | 0.003 | 0.026 |
| lh_entorhinal_volume | 7358357.89 | 2 | 243 | 17.889 | 0.000 | 0.000 |
| lh_fusiform_volume | 9995631.08 | 2 | 243 | 1.921 | 0.147 | 0.574 |
| lh_inferiorparietal_volume | 1881330.73 | 2 | 243 | 0.125 | 0.883 | 0.986 |
| lh_inferiortemporal_volume | 11022780.72 | 2 | 243 | 1.099 | 0.334 | 0.833 |
| lh_isthmuscingulate_volume | 16156.14 | 2 | 243 | 0.025 | 0.975 | 0.986 |
| lh_lateraloccipital_volume | 17546731.09 | 2 | 243 | 1.661 | 0.190 | 0.625 |
| lh_lateralorbitofrontal_volume | 1727608.08 | 2 | 243 | 0.593 | 0.553 | 0.910 |
| lh_lingual_volume | 7401412.64 | 2 | 243 | 2.258 | 0.105 | 0.478 |
| lh_medialorbitofrontal_volume | 5160764.04 | 2 | 243 | 3.407 | 0.033 | 0.229 |
| lh_middletemporal_volume | 5249571.52 | 2 | 243 | 0.642 | 0.527 | 0.904 |
| lh_parahippocampal_volume | 684501.76 | 2 | 243 | 2.892 | 0.056 | 0.328 |
| lh_paracentral_volume | 280460.39 | 2 | 243 | 0.280 | 0.756 | 0.986 |
| lh_parsopercularis_volume | 446108.62 | 2 | 243 | 0.145 | 0.865 | 0.986 |
| lh_parsorbitalis_volume | 344899.73 | 2 | 243 | 0.715 | 0.490 | 0.904 |
| lh_parstriangularis_volume | 217975.04 | 2 | 243 | 0.141 | 0.869 | 0.986 |
| lh_pericalcarine_volume | 4934524.96 | 2 | 243 | 8.343 | <0.001 | 0.005 |
| lh_postcentral_volume | 5650488.94 | 2 | 243 | 0.828 | 0.437 | 0.897 |
| lh_posteriorcingulate_volume | 15743.49 | 2 | 243 | 0.021 | 0.979 | 0.986 |
| lh_precentral_volume | 17445914.68 | 2 | 243 | 1.955 | 0.142 | 0.574 |
| lh_precuneus_volume | 5138898.42 | 2 | 243 | 0.652 | 0.521 | 0.904 |
| lh_rostralanteriorcingulate_volume | 1118017.92 | 2 | 243 | 1.276 | 0.279 | 0.833 |
| lh_rostralmiddlefrontal_volume | 16420069.72 | 2 | 243 | 0.682 | 0.506 | 0.904 |
| lh_superiorfrontal_volume | 13177556.43 | 2 | 243 | 0.475 | 0.622 | 0.956 |
| lh_superiorparietal_volume | 12165717.39 | 2 | 243 | 0.698 | 0.498 | 0.904 |
| lh_superiortemporal_volume | 1140789.28 | 2 | 243 | 0.149 | 0.861 | 0.986 |
| lh_supramarginal_volume | 1380729.87 | 2 | 243 | 0.097 | 0.908 | 0.986 |
| lh_frontalpole_volume | 55521.88 | 2 | 243 | 0.431 | 0.650 | 0.956 |
| lh_temporalpole_volume | 1372303.77 | 2 | 243 | 1.766 | 0.171 | 0.595 |
| lh_transversetemporal_volume | 14508.85 | 2 | 243 | 0.122 | 0.886 | 0.986 |
| lh_insula_volume | 811271.01 | 2 | 243 | 0.700 | 0.497 | 0.904 |
| rh_bankssts_volume | 56817.40 | 2 | 243 | 0.129 | 0.879 | 0.986 |
| rh_caudalanteriorcingulate_volume | 243179.90 | 2 | 243 | 0.358 | 0.699 | 0.956 |
| rh_caudalmiddlefrontal_volume | 4511606.99 | 2 | 243 | 0.931 | 0.394 | 0.874 |
| rh_cuneus_volume | 4742974.44 | 2 | 243 | 4.664 | 0.010 | 0.088 |
| rh_entorhinal_volume | 808323.06 | 2 | 243 | 2.324 | 0.098 | 0.478 |
| rh_fusiform_volume | 1668800.18 | 2 | 243 | 0.319 | 0.727 | 0.977 |
| rh_inferiorparietal_volume | 23930607.65 | 2 | 243 | 1.166 | 0.312 | 0.833 |
| rh_inferiortemporal_volume | 3374100.18 | 2 | 243 | 0.449 | 0.638 | 0.956 |
| rh_isthmuscingulate_volume | 522082.35 | 2 | 243 | 1.038 | 0.354 | 0.833 |
| rh_lateraloccipital_volume | 13009783.29 | 2 | 243 | 1.358 | 0.258 | 0.812 |
| rh_lateralorbitofrontal_volume | 3089089.81 | 2 | 243 | 1.103 | 0.332 | 0.833 |
| rh_lingual_volume | 4047715.24 | 2 | 243 | 1.071 | 0.343 | 0.833 |
| rh_medialorbitofrontal_volume | 2521518.83 | 2 | 243 | 1.764 | 0.172 | 0.595 |
| rh_middletemporal_volume | 6503216.07 | 2 | 243 | 0.827 | 0.438 | 0.897 |
| rh_parahippocampal_volume | 247085.57 | 2 | 243 | 1.035 | 0.356 | 0.833 |
| rh_paracentral_volume | 114301.49 | 2 | 243 | 0.127 | 0.881 | 0.986 |
| rh_parsopercularis_volume | 252013.29 | 2 | 243 | 0.125 | 0.883 | 0.986 |
| rh_parsorbitalis_volume | 974525.72 | 2 | 243 | 1.950 | 0.143 | 0.574 |
| rh_parstriangularis_volume | 234063.19 | 2 | 243 | 0.089 | 0.915 | 0.986 |
| rh_pericalcarine_volume | 1739950.91 | 2 | 243 | 2.819 | 0.060 | 0.328 |
| rh_postcentral_volume | 296852.35 | 2 | 243 | 0.047 | 0.954 | 0.986 |
| rh_posteriorcingulate_volume | 68009.00 | 2 | 243 | 0.082 | 0.921 | 0.986 |
| rh_precentral_volume | 668064.50 | 2 | 243 | 0.068 | 0.934 | 0.986 |
| rh_precuneus_volume | 8362828.01 | 2 | 243 | 0.945 | 0.389 | 0.874 |
| rh_rostralanteriorcingulate_volume | 241477.60 | 2 | 243 | 0.520 | 0.595 | 0.956 |
| rh_rostralmiddlefrontal_volume | 9068786.61 | 2 | 243 | 0.374 | 0.688 | 0.956 |
| rh_superiorfrontal_volume | 23185987.59 | 2 | 243 | 0.839 | 0.433 | 0.897 |
| rh_superiorparietal_volume | 11580450.22 | 2 | 243 | 0.732 | 0.481 | 0.904 |
| rh_superiortemporal_volume | 3410103.80 | 2 | 243 | 0.637 | 0.529 | 0.904 |
| rh_supramarginal_volume | 153160.58 | 2 | 243 | 0.014 | 0.986 | 0.986 |
| rh_frontalpole_volume | 101347.57 | 2 | 243 | 0.589 | 0.555 | 0.910 |
| rh_temporalpole_volume | 1353882.53 | 2 | 243 | 1.750 | 0.174 | 0.595 |
| rh_transversetemporal_volume | 62274.47 | 2 | 243 | 1.063 | 0.346 | 0.833 |
| rh_insula_volume | 73606.56 | 2 | 243 | 0.059 | 0.942 | 0.986 |
| Left-Thalamus | 3657540.16 | 2 | 243 | 2.837 | 0.059 | 0.328 |
| Left-Caudate | 2039141.35 | 2 | 243 | 4.507 | 0.011 | 0.092 |
| Left-Putamen | 53050.59 | 2 | 243 | 0.062 | 0.939 | 0.986 |
| Left-Pallidum | 38449.96 | 2 | 243 | 0.360 | 0.697 | 0.956 |
| Left-Hippocampus | 2787784.73 | 2 | 243 | 6.439 | 0.002 | 0.023 |
| Left-Amygdala | 889240.29 | 2 | 243 | 7.795 | 0.000 | 0.007 |
| Left-Accumbens-area | 1644.61 | 2 | 243 | 0.077 | 0.926 | 0.986 |
| Right-Thalamus | 8533543.99 | 2 | 243 | 8.581 | 0.000 | 0.005 |
| Right-Caudate | 1642466.66 | 2 | 243 | 3.562 | 0.029 | 0.214 |
| Right-Putamen | 396890.04 | 2 | 243 | 0.488 | 0.614 | 0.956 |
| Right-Pallidum | 131710.12 | 2 | 243 | 1.241 | 0.290 | 0.833 |
| Right-Hippocampus | 4092688.03 | 2 | 243 | 9.939 | 0.000 | 0.002 |
| Right-Amygdala | 594817.40 | 2 | 243 | 6.017 | 0.003 | 0.026 |
| Right-Accumbens-area | 38737.37 | 2 | 243 | 2.260 | 0.105 | 0.478 |

ROI = regions of interest

FDR = false discovery rate

| **Table S7 The result of post-hoc tests among the raw data, TS-corrected data and ComBat-corrected data** | | | | | |
| --- | --- | --- | --- | --- | --- |
| Raw vs TS |  |  |  |  |  |
|  |  |  | Confidence interval | |  |
| ROI | meandiff | FWE p | lower | upper | FDR p |
| lh_bankssts_volume | -19.36 | 0.901 | -123.59 | 84.88 | 1.000 |
| lh_caudalanteriorcingulate_volume | -14.10 | 0.926 | -102.94 | 74.75 | 1.000 |
| lh_caudalmiddlefrontal_volume | 87.75 | 0.735 | -187.50 | 362.99 | 1.000 |
| lh_cuneus_volume | 122.97 | 0.036 | 6.43 | 239.52 | 0.419 |
| lh_entorhinal_volume | -158.27 | <0.001 | -234.88 | -81.66 | <0.001 |
| lh_fusiform_volume | -155.84 | 0.372 | -428.34 | 116.67 | 1.000 |
| lh_inferiorparietal_volume | -29.13 | 0.988 | -493.39 | 435.13 | 1.000 |
| lh_inferiortemporal_volume | -133.70 | 0.685 | -512.01 | 244.60 | 1.000 |
| lh_isthmuscingulate_volume | 9.12 | 0.973 | -86.12 | 104.35 | 1.000 |
| lh_lateraloccipital_volume | 192.28 | 0.476 | -195.96 | 580.52 | 1.000 |
| lh_lateralorbitofrontal_volume | -67.81 | 0.715 | -271.62 | 136.00 | 1.000 |
| lh_lingual_volume | 123.65 | 0.372 | -92.60 | 339.91 | 1.000 |
| lh_medialorbitofrontal_volume | -146.26 | 0.052 | -293.29 | 0.76 | 0.469 |
| lh_middletemporal_volume | -42.59 | 0.954 | -384.29 | 299.11 | 1.000 |
| lh_parahippocampal_volume | -52.01 | 0.090 | -110.12 | 6.11 | 0.569 |
| lh_paracentral_volume | 33.32 | 0.790 | -86.25 | 152.89 | 1.000 |
| lh_parsopercularis_volume | -44.24 | 0.874 | -253.83 | 165.36 | 1.000 |
| lh_parsorbitalis_volume | -26.06 | 0.742 | -109.04 | 56.92 | 1.000 |
| lh_parstriangularis_volume | -13.20 | 0.976 | -161.89 | 135.49 | 1.000 |
| lh_pericalcarine_volume | 118.90 | 0.007 | 27.03 | 210.76 | 0.141 |
| lh_postcentral_volume | 154.58 | 0.476 | -157.50 | 466.67 | 1.000 |
| lh_posteriorcingulate_volume | 0.80 | 1.000 | -102.52 | 104.12 | 1.000 |
| lh_precentral_volume | 252.39 | 0.221 | -104.45 | 609.23 | 1.000 |
| lh_precuneus_volume | 108.92 | 0.726 | -226.43 | 444.27 | 1.000 |
| lh_rostralanteriorcingulate_volume | -63.03 | 0.383 | -174.82 | 48.76 | 1.000 |
| lh_rostralmiddlefrontal_volume | -163.69 | 0.789 | -749.59 | 422.22 | 1.000 |
| lh_superiorfrontal_volume | -195.11 | 0.747 | -824.33 | 434.11 | 1.000 |
| lh_superiorparietal_volume | 215.11 | 0.569 | -283.70 | 713.93 | 1.000 |
| lh_superiortemporal_volume | 64.58 | 0.890 | -265.50 | 394.66 | 1.000 |
| lh_supramarginal_volume | 79.32 | 0.910 | -371.61 | 530.24 | 1.000 |
| lh_frontalpole_volume | 16.85 | 0.627 | -26.03 | 59.73 | 1.000 |
| lh_temporalpole_volume | -73.29 | 0.232 | -178.59 | 32.00 | 1.000 |
| lh_transversetemporal_volume | 1.65 | 0.995 | -39.62 | 42.91 | 1.000 |
| lh_insula_volume | 50.95 | 0.621 | -77.60 | 179.50 | 1.000 |
| rh_bankssts_volume | -2.42 | 0.997 | -81.82 | 76.99 | 1.000 |
| rh_caudalanteriorcingulate_volume | -24.54 | 0.828 | -123.01 | 73.92 | 1.000 |
| rh_caudalmiddlefrontal_volume | 133.82 | 0.457 | -129.07 | 396.71 | 1.000 |
| rh_cuneus_volume | 112.77 | 0.072 | -7.69 | 233.23 | 0.493 |
| rh_entorhinal_volume | -43.31 | 0.319 | -113.75 | 27.14 | 1.000 |
| rh_fusiform_volume | -36.65 | 0.947 | -309.92 | 236.62 | 1.000 |
| rh_inferiorparietal_volume | -216.57 | 0.616 | -757.70 | 324.56 | 1.000 |
| rh_inferiortemporal_volume | -67.93 | 0.878 | -395.33 | 259.47 | 1.000 |
| rh_isthmuscingulate_volume | 34.20 | 0.610 | -50.50 | 118.90 | 1.000 |
| rh_lateraloccipital_volume | 188.23 | 0.457 | -181.48 | 557.94 | 1.000 |
| rh_lateralorbitofrontal_volume | -88.40 | 0.553 | -288.33 | 111.53 | 1.000 |
| rh_lingual_volume | 76.02 | 0.723 | -156.17 | 308.21 | 1.000 |
| rh_medialorbitofrontal_volume | -104.21 | 0.201 | -247.03 | 38.60 | 1.000 |
| rh_middletemporal_volume | 0.06 | 1.000 | -334.97 | 335.09 | 1.000 |
| rh_parahippocampal_volume | -32.50 | 0.392 | -90.88 | 25.87 | 1.000 |
| rh_paracentral_volume | -24.32 | 0.870 | -137.73 | 89.09 | 1.000 |
| rh_parsopercularis_volume | 31.99 | 0.898 | -137.76 | 201.73 | 1.000 |
| rh_parsorbitalis_volume | -46.85 | 0.394 | -131.30 | 37.60 | 1.000 |
| rh_parstriangularis_volume | -6.65 | 0.996 | -200.47 | 187.16 | 1.000 |
| rh_pericalcarine_volume | 67.07 | 0.214 | -26.77 | 160.91 | 1.000 |
| rh_postcentral_volume | 12.71 | 0.995 | -288.42 | 313.83 | 1.000 |
| rh_posteriorcingulate_volume | 18.29 | 0.917 | -90.22 | 126.80 | 1.000 |
| rh_precentral_volume | 18.78 | 0.992 | -354.45 | 392.01 | 1.000 |
| rh_precuneus_volume | 164.42 | 0.523 | -190.99 | 519.84 | 1.000 |
| rh_rostralanteriorcingulate_volume | -30.03 | 0.662 | -111.45 | 51.39 | 1.000 |
| rh_rostralmiddlefrontal_volume | -48.89 | 0.979 | -636.70 | 538.93 | 1.000 |
| rh_superiorfrontal_volume | -215.82 | 0.699 | -843.95 | 412.31 | 1.000 |
| rh_superiorparietal_volume | 210.42 | 0.552 | -264.84 | 685.68 | 1.000 |
| rh_superiortemporal_volume | 107.54 | 0.632 | -168.92 | 384.01 | 1.000 |
| rh_supramarginal_volume | 28.17 | 0.985 | -372.09 | 428.42 | 1.000 |
| rh_frontalpole_volume | 22.73 | 0.529 | -26.83 | 72.28 | 1.000 |
| rh_temporalpole_volume | -79.50 | 0.178 | -184.56 | 25.56 | 1.000 |
| rh_transversetemporal_volume | 10.93 | 0.649 | -17.99 | 39.85 | 1.000 |
| rh_insula_volume | 11.55 | 0.977 | -121.41 | 144.51 | 1.000 |
| Left-Thalamus | -99.00 | 0.201 | -234.63 | 36.64 | 1.000 |
| Left-Caudate | 82.70 | 0.042 | 2.35 | 163.04 | 0.429 |
| Left-Putamen | 6.75 | 0.989 | -103.35 | 116.85 | 1.000 |
| Left-Pallidum | 2.07 | 0.992 | -36.94 | 41.08 | 1.000 |
| Left-Hippocampus | -87.55 | 0.025 | -166.15 | -8.96 | 0.336 |
| Left-Amygdala | -47.81 | 0.015 | -88.16 | -7.47 | 0.249 |
| Left-Accumbens-area | 0.44 | 0.998 | -16.97 | 17.85 | 1.000 |
| Right-Thalamus | -170.05 | 0.002 | -289.16 | -50.93 | 0.071 |
| Right-Caudate | 77.80 | 0.063 | -3.32 | 158.92 | 0.473 |
| Right-Putamen | -16.60 | 0.931 | -124.37 | 91.18 | 1.000 |
| Right-Pallidum | 25.39 | 0.277 | -13.53 | 64.31 | 1.000 |
| Right-Hippocampus | -108.74 | 0.003 | -185.40 | -32.09 | 0.071 |
| Right-Amygdala | -36.02 | 0.063 | -73.58 | 1.54 | 0.473 |
| Right-Accumbens-area | -10.33 | 0.268 | -25.97 | 5.31 | 1.000 |
| ComBat vs TS |  |  |  |  |  |
|  |  |  | Confidence interval | |  |
| ROI | meandiff | FWE p | lower | upper | FDR p |
| lh_bankssts_volume | -40.68 | 0.646 | -147.88 | 66.52 | 0.982 |
| lh_caudalanteriorcingulate_volume | -19.80 | 0.867 | -111.18 | 71.58 | 0.997 |
| lh_caudalmiddlefrontal_volume | 89.34 | 0.739 | -193.75 | 372.43 | 0.997 |
| lh_cuneus_volume | 169.91 | 0.003 | 50.04 | 289.77 | 0.027 |
| .hjlh_entorhinal_volume | -181.91 | <0.001 | -260.71 | -103.12 | <0.001 |
| lh_fusiform_volume | -226.98 | 0.139 | -507.25 | 53.29 | 0.570 |
| lh_inferiorparietal_volume | -99.35 | 0.877 | -576.84 | 378.14 | 0.997 |
| lh_inferiortemporal_volume | -244.88 | 0.302 | -633.97 | 144.20 | 0.862 |
| lh_isthmuscingulate_volume | 5.27 | 0.991 | -92.69 | 103.22 | 0.997 |
| lh_lateraloccipital_volume | 304.89 | 0.173 | -94.42 | 704.20 | 0.674 |
| lh_lateralorbitofrontal_volume | -93.21 | 0.550 | -302.82 | 116.41 | 0.941 |
| lh_lingual_volume | 198.31 | 0.092 | -24.11 | 420.73 | 0.470 |
| lh_medialorbitofrontal_volume | -138.85 | 0.080 | -290.06 | 12.36 | 0.466 |
| lh_middletemporal_volume | -164.50 | 0.515 | -515.94 | 186.94 | 0.941 |
| lh_parahippocampal_volume | -52.08 | 0.102 | -111.85 | 7.69 | 0.486 |
| lh_paracentral_volume | 33.30 | 0.801 | -89.67 | 156.28 | 0.997 |
| lh_parsopercularis_volume | -39.04 | 0.905 | -254.61 | 176.52 | 0.997 |
| lh_parsorbitalis_volume | -42.95 | 0.465 | -128.29 | 42.40 | 0.941 |
| lh_parstriangularis_volume | -34.38 | 0.858 | -187.30 | 118.55 | 0.997 |
| lh_pericalcarine_volume | 155.54 | <0.001 | 61.06 | 250.02 | 0.008 |
| lh_postcentral_volume | 143.22 | 0.547 | -177.76 | 464.20 | 0.941 |
| lh_posteriorcingulate_volume | -7.69 | 0.984 | -113.95 | 98.58 | 0.997 |
| lh_precentral_volume | 273.04 | 0.189 | -93.97 | 640.05 | 0.703 |
| lh_precuneus_volume | 163.66 | 0.506 | -181.24 | 508.57 | 0.941 |
| lh_rostralanteriorcingulate_volume | -69.86 | 0.328 | -184.83 | 45.12 | 0.862 |
| lh_rostralmiddlefrontal_volume | -298.83 | 0.475 | -901.43 | 303.77 | 0.941 |
| lh_superiorfrontal_volume | -253.77 | 0.628 | -900.92 | 393.38 | 0.982 |
| lh_superiorparietal_volume | 223.92 | 0.562 | -289.11 | 736.94 | 0.941 |
| lh_superiortemporal_volume | 69.78 | 0.880 | -269.70 | 409.26 | 0.997 |
| lh_supramarginal_volume | 66.06 | 0.940 | -397.71 | 529.84 | 0.997 |
| lh_frontalpole_volume | 6.65 | 0.933 | -37.45 | 50.75 | 0.997 |
| lh_temporalpole_volume | -74.12 | 0.243 | -182.42 | 34.18 | 0.862 |
| lh_transversetemporal_volume | 8.47 | 0.886 | -33.96 | 50.91 | 0.997 |
| lh_insula_volume | 61.51 | 0.519 | -70.70 | 193.72 | 0.941 |
| rh_bankssts_volume | -16.48 | 0.884 | -98.15 | 65.20 | 0.997 |
| rh_caudalanteriorcingulate_volume | -35.32 | 0.692 | -136.59 | 65.95 | 0.997 |
| rh_caudalmiddlefrontal_volume | 133.38 | 0.479 | -137.01 | 403.76 | 0.941 |
| rh_cuneus_volume | 154.26 | 0.010 | 30.36 | 278.15 | 0.090 |
| rh_entorhinal_volume | -64.88 | 0.090 | -137.33 | 7.57 | 0.470 |
| rh_fusiform_volume | -95.13 | 0.707 | -376.19 | 185.92 | 0.997 |
| rh_inferiorparietal_volume | -357.83 | 0.287 | -914.38 | 198.72 | 0.862 |
| rh_inferiortemporal_volume | -135.92 | 0.610 | -472.65 | 200.81 | 0.981 |
| rh_isthmuscingulate_volume | 52.32 | 0.336 | -34.79 | 139.43 | 0.862 |
| rh_lateraloccipital_volume | 254.84 | 0.258 | -125.41 | 635.09 | 0.862 |
| rh_lateralorbitofrontal_volume | -125.54 | 0.324 | -331.17 | 80.09 | 0.862 |
| rh_lingual_volume | 148.80 | 0.310 | -90.01 | 387.60 | 0.862 |
| rh_medialorbitofrontal_volume | -94.31 | 0.288 | -241.19 | 52.58 | 0.862 |
| rh_middletemporal_volume | -164.95 | 0.500 | -509.53 | 179.62 | 0.941 |
| rh_parahippocampal_volume | -29.69 | 0.477 | -89.73 | 30.35 | 0.941 |
| rh_paracentral_volume | -11.36 | 0.972 | -128.00 | 105.28 | 0.997 |
| rh_parsopercularis_volume | 31.10 | 0.908 | -143.48 | 205.69 | 0.997 |
| rh_parsorbitalis_volume | -71.44 | 0.131 | -158.30 | 15.41 | 0.564 |
| rh_parstriangularis_volume | -34.05 | 0.915 | -233.39 | 165.29 | 0.997 |
| rh_pericalcarine_volume | 93.94 | 0.058 | -2.57 | 190.45 | 0.373 |
| rh_postcentral_volume | 39.70 | 0.951 | -270.01 | 349.41 | 0.997 |
| rh_posteriorcingulate_volume | 12.95 | 0.960 | -98.65 | 124.56 | 0.997 |
| rh_precentral_volume | 59.50 | 0.930 | -324.36 | 443.37 | 0.997 |
| rh_precuneus_volume | 196.94 | 0.416 | -168.60 | 562.49 | 0.941 |
| rh_rostralanteriorcingulate_volume | -31.82 | 0.646 | -115.56 | 51.92 | 0.982 |
| rh_rostralmiddlefrontal_volume | -214.25 | 0.683 | -818.82 | 390.31 | 0.997 |
| rh_superiorfrontal_volume | -351.67 | 0.408 | -997.70 | 294.36 | 0.941 |
| rh_superiorparietal_volume | 217.92 | 0.548 | -270.88 | 706.72 | 0.941 |
| rh_superiortemporal_volume | 123.99 | 0.562 | -160.36 | 408.33 | 0.941 |
| rh_supramarginal_volume | 13.73 | 0.997 | -397.93 | 425.39 | 0.997 |
| rh_frontalpole_volume | 8.73 | 0.915 | -42.24 | 59.70 | 0.997 |
| rh_temporalpole_volume | -63.45 | 0.353 | -171.50 | 44.61 | 0.877 |
| rh_transversetemporal_volume | 18.28 | 0.320 | -11.47 | 48.02 | 0.862 |
| rh_insula_volume | 19.93 | 0.938 | -116.82 | 156.68 | 0.997 |
| Left-Thalamus | -135.47 | 0.059 | -274.97 | 4.03 | 0.373 |
| Left-Caudate | 96.22 | 0.018 | 13.58 | 178.85 | 0.144 |
| Left-Putamen | -10.23 | 0.976 | -123.47 | 103.00 | 0.997 |
| Left-Pallidum | -11.51 | 0.779 | -51.64 | 28.61 | 0.997 |
| Left-Hippocampus | -117.78 | 0.002 | -198.61 | -36.94 | 0.023 |
| Left-Amygdala | -67.21 | 0.000 | -108.70 | -25.72 | 0.008 |
| Left-Accumbens-area | -2.37 | 0.948 | -20.28 | 15.53 | 0.997 |
| Right-Thalamus | -196.20 | 0.001 | -318.71 | -73.68 | 0.008 |
| Right-Caudate | 83.45 | 0.050 | 0.02 | 166.88 | 0.372 |
| Right-Putamen | -46.23 | 0.591 | -157.07 | 64.62 | 0.968 |
| Right-Pallidum | 18.21 | 0.535 | -21.82 | 58.24 | 0.941 |
| Right-Hippocampus | -141.42 | <0.001 | -220.26 | -62.59 | 0.004 |
| Right-Amygdala | -55.98 | 0.002 | -94.61 | -17.35 | 0.023 |
| Right-Accumbens-area | -13.88 | 0.107 | -29.96 | 2.21 | 0.486 |
| ComBat vs Raw |  |  |  |  |  |
|  |  |  | Confidence interval | |  |
| ROI | Mean difference | FWE p | lower | upper | FDR p |
| lh_bankssts_volume | -21.32 | 0.887 | -128.52 | 85.88 | 1.000 |
| lh_caudalanteriorcingulate_volume | -5.70 | 0.988 | -97.08 | 85.67 | 1.000 |
| lh_caudalmiddlefrontal_volume | 1.60 | 1.000 | -281.49 | 284.68 | 1.000 |
| lh_cuneus_volume | 46.94 | 0.628 | -72.93 | 166.80 | 1.000 |
| lh_entorhinal_volume | -23.65 | 0.761 | -102.44 | 55.15 | 1.000 |
| lh_fusiform_volume | -71.14 | 0.822 | -351.42 | 209.13 | 1.000 |
| lh_inferiorparietal_volume | -70.22 | 0.937 | -547.71 | 407.26 | 1.000 |
| lh_inferiortemporal_volume | -111.18 | 0.781 | -500.27 | 277.91 | 1.000 |
| lh_isthmuscingulate_volume | -3.85 | 0.995 | -101.80 | 94.11 | 1.000 |
| lh_lateraloccipital_volume | 112.61 | 0.786 | -286.70 | 511.91 | 1.000 |
| lh_lateralorbitofrontal_volume | -25.40 | 0.956 | -235.02 | 184.22 | 1.000 |
| lh_lingual_volume | 74.66 | 0.711 | -147.76 | 297.07 | 1.000 |
| lh_medialorbitofrontal_volume | 7.41 | 0.993 | -143.80 | 158.63 | 1.000 |
| lh_middletemporal_volume | -121.91 | 0.694 | -473.34 | 229.53 | 1.000 |
| lh_parahippocampal_volume | -0.08 | 1.000 | -59.84 | 59.69 | 1.000 |
| lh_paracentral_volume | -0.02 | 1.000 | -122.99 | 122.96 | 1.000 |
| lh_parsopercularis_volume | 5.19 | 0.998 | -210.37 | 220.76 | 1.000 |
| lh_parsorbitalis_volume | -16.88 | 0.888 | -102.23 | 68.46 | 1.000 |
| lh_parstriangularis_volume | -21.18 | 0.944 | -174.10 | 131.75 | 1.000 |
| lh_pericalcarine_volume | 36.65 | 0.634 | -57.83 | 131.13 | 1.000 |
| lh_postcentral_volume | -11.36 | 0.996 | -332.35 | 309.62 | 1.000 |
| lh_posteriorcingulate_volume | -8.49 | 0.981 | -114.75 | 97.78 | 1.000 |
| lh_precentral_volume | 20.65 | 0.990 | -346.36 | 387.66 | 1.000 |
| lh_precuneus_volume | 54.74 | 0.926 | -290.16 | 399.65 | 1.000 |
| lh_rostralanteriorcingulate_volume | -6.82 | 0.989 | -121.80 | 108.15 | 1.000 |
| lh_rostralmiddlefrontal_volume | -135.14 | 0.859 | -737.74 | 467.46 | 1.000 |
| lh_superiorfrontal_volume | -58.67 | 0.975 | -705.82 | 588.48 | 1.000 |
| lh_superiorparietal_volume | 8.80 | 0.999 | -504.22 | 521.83 | 1.000 |
| lh_superiortemporal_volume | 5.20 | 0.999 | -334.28 | 344.68 | 1.000 |
| lh_supramarginal_volume | -13.25 | 0.998 | -477.03 | 450.52 | 1.000 |
| lh_frontalpole_volume | -10.20 | 0.850 | -54.30 | 33.90 | 1.000 |
| lh_temporalpole_volume | -0.82 | 1.000 | -109.12 | 107.47 | 1.000 |
| lh_transversetemporal_volume | 6.83 | 0.924 | -35.61 | 49.27 | 1.000 |
| lh_insula_volume | 10.56 | 0.981 | -121.65 | 142.77 | 1.000 |
| rh_bankssts_volume | -14.06 | 0.914 | -95.73 | 67.61 | 1.000 |
| rh_caudalanteriorcingulate_volume | -10.78 | 0.966 | -112.05 | 90.49 | 1.000 |
| rh_caudalmiddlefrontal_volume | -0.44 | 1.000 | -270.82 | 269.94 | 1.000 |
| rh_cuneus_volume | 41.49 | 0.712 | -82.40 | 165.38 | 1.000 |
| rh_entorhinal_volume | -21.57 | 0.764 | -94.02 | 50.88 | 1.000 |
| rh_fusiform_volume | -58.48 | 0.877 | -339.54 | 222.57 | 1.000 |
| rh_inferiorparietal_volume | -141.26 | 0.823 | -697.81 | 415.29 | 1.000 |
| rh_inferiortemporal_volume | -67.99 | 0.884 | -404.72 | 268.74 | 1.000 |
| rh_isthmuscingulate_volume | 18.12 | 0.877 | -68.99 | 105.23 | 1.000 |
| rh_lateraloccipital_volume | 66.61 | 0.911 | -313.64 | 446.86 | 1.000 |
| rh_lateralorbitofrontal_volume | -37.14 | 0.906 | -242.77 | 168.49 | 1.000 |
| rh_lingual_volume | 72.77 | 0.755 | -166.03 | 311.58 | 1.000 |
| rh_medialorbitofrontal_volume | 9.91 | 0.986 | -136.98 | 156.79 | 1.000 |
| rh_middletemporal_volume | -165.01 | 0.500 | -509.59 | 179.57 | 1.000 |
| rh_parahippocampal_volume | 2.81 | 0.993 | -57.23 | 62.85 | 1.000 |
| rh_paracentral_volume | 12.96 | 0.963 | -103.68 | 129.60 | 1.000 |
| rh_parsopercularis_volume | -0.88 | 1.000 | -175.47 | 173.70 | 1.000 |
| rh_parsorbitalis_volume | -24.59 | 0.784 | -111.45 | 62.26 | 1.000 |
| rh_parstriangularis_volume | -27.40 | 0.944 | -226.73 | 171.94 | 1.000 |
| rh_pericalcarine_volume | 26.87 | 0.791 | -69.65 | 123.38 | 1.000 |
| rh_postcentral_volume | 26.99 | 0.977 | -282.71 | 336.70 | 1.000 |
| rh_posteriorcingulate_volume | -5.33 | 0.993 | -116.93 | 106.27 | 1.000 |
| rh_precentral_volume | 40.72 | 0.966 | -343.15 | 424.59 | 1.000 |
| rh_precuneus_volume | 32.52 | 0.976 | -333.02 | 398.06 | 1.000 |
| rh_rostralanteriorcingulate_volume | -1.79 | 0.999 | -85.53 | 81.95 | 1.000 |
| rh_rostralmiddlefrontal_volume | -165.36 | 0.797 | -769.93 | 439.20 | 1.000 |
| rh_superiorfrontal_volume | -135.85 | 0.874 | -781.88 | 510.17 | 1.000 |
| rh_superiorparietal_volume | 7.50 | 0.999 | -481.30 | 496.30 | 1.000 |
| rh_superiortemporal_volume | 16.44 | 0.990 | -267.90 | 300.78 | 1.000 |
| rh_supramarginal_volume | -14.44 | 0.996 | -426.10 | 397.22 | 1.000 |
| rh_frontalpole_volume | -14.00 | 0.796 | -64.96 | 36.97 | 1.000 |
| rh_temporalpole_volume | 16.05 | 0.935 | -92.01 | 124.10 | 1.000 |
| rh_transversetemporal_volume | 7.35 | 0.831 | -22.39 | 37.09 | 1.000 |
| rh_insula_volume | 8.38 | 0.989 | -128.37 | 145.13 | 1.000 |
| Left-Thalamus | -36.47 | 0.813 | -175.98 | 103.03 | 1.000 |
| Left-Caudate | 13.52 | 0.922 | -69.11 | 96.15 | 1.000 |
| Left-Putamen | -16.99 | 0.934 | -130.22 | 96.25 | 1.000 |
| Left-Pallidum | -13.58 | 0.707 | -53.71 | 26.54 | 1.000 |
| Left-Hippocampus | -30.22 | 0.655 | -111.06 | 50.62 | 1.000 |
| Left-Amygdala | -19.40 | 0.516 | -60.89 | 22.09 | 1.000 |
| Left-Accumbens-area | -2.81 | 0.928 | -20.72 | 15.09 | 1.000 |
| Right-Thalamus | -26.15 | 0.871 | -148.66 | 96.36 | 1.000 |
| Right-Caudate | 5.65 | 0.986 | -77.78 | 89.08 | 1.000 |
| Right-Putamen | -29.63 | 0.805 | -140.48 | 81.22 | 1.000 |
| Right-Pallidum | -7.19 | 0.907 | -47.21 | 32.84 | 1.000 |
| Right-Hippocampus | -32.68 | 0.594 | -111.51 | 46.16 | 1.000 |
| Right-Amygdala | -19.96 | 0.446 | -58.59 | 18.67 | 1.000 |
| Right-Accumbens-area | -3.55 | 0.863 | -19.63 | 12.54 | 1.000 |

ROI = regions of interest

FWE = family wise error

FDR = false discovery rate

**Table S8** **The results of the difference between ADHD and TD by using raw data**

|  |  | Confidence interval | |  |  |  |  |  |
| --- | --- | --- | --- | --- | --- | --- | --- | --- |
| ROI | beta | 2.50% | 97.50% | t value | d.f. | p | FDR p | r2 |
| lh_bankssts_volume | -0.115 | -0.246 | 0.015 | -1.731 | 279.281 | 0.085 | 0.347 | 0.085 |
| lh_caudalanteriorcingulate_volume | 0.017 | -0.117 | 0.150 | 0.242 | 300.500 | 0.809 | 0.873 | 0.064 |
| lh_caudalmiddlefrontal_volume | -0.029 | -0.155 | 0.097 | -0.453 | 292.768 | 0.651 | 0.769 | 0.161 |
| lh_cuneus_volume | 0.078 | -0.053 | 0.210 | 1.166 | 275.021 | 0.244 | 0.542 | 0.106 |
| lh_entorhinal_volume | -0.029 | -0.156 | 0.099 | -0.439 | 266.380 | 0.661 | 0.769 | 0.039 |
| lh_fusiform_volume | -0.105 | -0.219 | 0.009 | -1.807 | 283.096 | 0.072 | 0.327 | 0.278 |
| lh_inferiorparietal_volume | -0.032 | -0.147 | 0.082 | -0.550 | 281.702 | 0.583 | 0.769 | 0.238 |
| lh_inferiortemporal_volume | -0.148 | -0.259 | -0.037 | -2.619 | 291.827 | 0.009 | 0.109 | 0.297 |
| lh_isthmuscingulate_volume | -0.055 | -0.182 | 0.071 | -0.863 | 282.235 | 0.389 | 0.668 | 0.169 |
| lh_lateraloccipital_volume | 0.057 | -0.059 | 0.173 | 0.967 | 282.667 | 0.334 | 0.637 | 0.194 |
| lh_lateralorbitofrontal_volume | -0.226 | -0.326 | -0.126 | -4.436 | 280.089 | 0.000 | 0.001 | 0.326 |
| lh_lingual_volume | 0.010 | -0.121 | 0.140 | 0.144 | 287.020 | 0.885 | 0.931 | 0.133 |
| lh_medialorbitofrontal_volume | -0.035 | -0.146 | 0.075 | -0.627 | 281.148 | 0.531 | 0.769 | 0.309 |
| lh_middletemporal_volume | -0.153 | -0.262 | -0.044 | -2.756 | 278.002 | 0.006 | 0.085 | 0.266 |
| lh_parahippocampal_volume | -0.094 | -0.224 | 0.035 | -1.425 | 283.005 | 0.155 | 0.481 | 0.097 |
| lh_paracentral_volume | -0.003 | -0.127 | 0.122 | -0.044 | 286.707 | 0.965 | 0.965 | 0.163 |
| lh_parsopercularis_volume | -0.057 | -0.188 | 0.073 | -0.860 | 282.034 | 0.391 | 0.668 | 0.073 |
| lh_parsorbitalis_volume | -0.124 | -0.241 | -0.007 | -2.083 | 285.071 | 0.038 | 0.209 | 0.127 |
| lh_parstriangularis_volume | -0.078 | -0.204 | 0.048 | -1.206 | 279.575 | 0.229 | 0.523 | 0.094 |
| lh_pericalcarine_volume | 0.004 | -0.125 | 0.132 | 0.053 | 280.662 | 0.957 | 0.965 | 0.130 |
| lh_postcentral_volume | -0.058 | -0.180 | 0.064 | -0.925 | 289.515 | 0.356 | 0.649 | 0.191 |
| lh_posteriorcingulate_volume | -0.033 | -0.157 | 0.091 | -0.527 | 281.035 | 0.598 | 0.769 | 0.197 |
| lh_precentral_volume | -0.083 | -0.201 | 0.036 | -1.365 | 282.894 | 0.173 | 0.490 | 0.241 |
| lh_precuneus_volume | -0.137 | -0.244 | -0.030 | -2.521 | 286.133 | 0.012 | 0.125 | 0.346 |
| lh_rostralanteriorcingulate_volume | -0.042 | -0.162 | 0.078 | -0.688 | 285.944 | 0.492 | 0.749 | 0.211 |
| lh_rostralmiddlefrontal_volume | -0.062 | -0.182 | 0.057 | -1.018 | 280.380 | 0.310 | 0.630 | 0.134 |
| lh_superiorfrontal_volume | -0.011 | -0.124 | 0.102 | -0.190 | 289.591 | 0.849 | 0.904 | 0.305 |
| lh_superiorparietal_volume | -0.029 | -0.148 | 0.090 | -0.480 | 276.712 | 0.632 | 0.769 | 0.221 |
| lh_superiortemporal_volume | -0.081 | -0.206 | 0.045 | -1.264 | 279.613 | 0.207 | 0.523 | 0.141 |
| lh_supramarginal_volume | -0.143 | -0.265 | -0.021 | -2.303 | 279.024 | 0.022 | 0.139 | 0.210 |
| lh_frontalpole_volume | -0.030 | -0.158 | 0.097 | -0.465 | 272.341 | 0.642 | 0.769 | 0.094 |
| lh_temporalpole_volume | -0.121 | -0.244 | 0.003 | -1.916 | 272.853 | 0.056 | 0.272 | 0.119 |
| lh_transversetemporal_volume | -0.137 | -0.268 | -0.005 | -2.038 | 295.261 | 0.042 | 0.217 | 0.081 |
| lh_insula_volume | -0.147 | -0.268 | -0.027 | -2.393 | 266.848 | 0.017 | 0.139 | 0.213 |
| rh_bankssts_volume | -0.081 | -0.213 | 0.051 | -1.204 | 285.549 | 0.230 | 0.523 | 0.081 |
| rh_caudalanteriorcingulate_volume | -0.028 | -0.154 | 0.097 | -0.443 | 293.648 | 0.658 | 0.769 | 0.088 |
| rh_caudalmiddlefrontal_volume | 0.055 | -0.075 | 0.184 | 0.830 | 281.759 | 0.407 | 0.681 | 0.131 |
| rh_cuneus_volume | -0.026 | -0.147 | 0.096 | -0.412 | 284.590 | 0.681 | 0.776 | 0.191 |
| rh_entorhinal_volume | -0.016 | -0.146 | 0.114 | -0.244 | 265.657 | 0.808 | 0.873 | 0.064 |
| rh_fusiform_volume | -0.084 | -0.199 | 0.031 | -1.434 | 270.612 | 0.153 | 0.481 | 0.251 |
| rh_inferiorparietal_volume | -0.076 | -0.190 | 0.039 | -1.292 | 286.774 | 0.197 | 0.523 | 0.234 |
| rh_inferiortemporal_volume | -0.043 | -0.160 | 0.073 | -0.726 | 262.491 | 0.468 | 0.749 | 0.244 |
| rh_isthmuscingulate_volume | -0.004 | -0.131 | 0.123 | -0.059 | 284.299 | 0.953 | 0.965 | 0.143 |
| rh_lateraloccipital_volume | 0.055 | -0.070 | 0.180 | 0.862 | 275.317 | 0.389 | 0.668 | 0.158 |
| rh_lateralorbitofrontal_volume | -0.193 | -0.296 | -0.090 | -3.680 | 274.439 | 0.000 | 0.008 | 0.356 |
| rh_lingual_volume | -0.034 | -0.162 | 0.095 | -0.518 | 280.363 | 0.605 | 0.769 | 0.149 |
| rh_medialorbitofrontal_volume | -0.092 | -0.205 | 0.020 | -1.610 | 283.425 | 0.108 | 0.404 | 0.320 |
| rh_middletemporal_volume | -0.255 | -0.366 | -0.145 | -4.535 | 268.837 | 0.000 | 0.001 | 0.260 |
| rh_parahippocampal_volume | -0.092 | -0.220 | 0.036 | -1.414 | 284.921 | 0.158 | 0.481 | 0.082 |
| rh_paracentral_volume | -0.111 | -0.234 | 0.012 | -1.765 | 281.004 | 0.079 | 0.340 | 0.177 |
| rh_parsopercularis_volume | -0.111 | -0.241 | 0.018 | -1.683 | 285.001 | 0.094 | 0.365 | 0.105 |
| rh_parsorbitalis_volume | -0.172 | -0.290 | -0.054 | -2.850 | 275.043 | 0.005 | 0.085 | 0.130 |
| rh_parstriangularis_volume | -0.153 | -0.284 | -0.023 | -2.302 | 272.827 | 0.022 | 0.139 | 0.093 |
| rh_pericalcarine_volume | -0.062 | -0.186 | 0.062 | -0.983 | 285.766 | 0.327 | 0.637 | 0.180 |
| rh_postcentral_volume | -0.029 | -0.150 | 0.093 | -0.462 | 287.229 | 0.645 | 0.769 | 0.228 |
| rh_posteriorcingulate_volume | -0.028 | -0.151 | 0.095 | -0.451 | 280.612 | 0.652 | 0.769 | 0.211 |
| rh_precentral_volume | 0.032 | -0.093 | 0.156 | 0.498 | 286.775 | 0.619 | 0.769 | 0.163 |
| rh_precuneus_volume | -0.067 | -0.175 | 0.040 | -1.227 | 282.585 | 0.221 | 0.523 | 0.359 |
| rh_rostralanteriorcingulate_volume | -0.091 | -0.215 | 0.032 | -1.455 | 283.061 | 0.147 | 0.481 | 0.165 |
| rh_rostralmiddlefrontal_volume | -0.167 | -0.283 | -0.050 | -2.791 | 273.815 | 0.006 | 0.085 | 0.166 |
| rh_superiorfrontal_volume | -0.084 | -0.203 | 0.035 | -1.383 | 290.198 | 0.168 | 0.490 | 0.235 |
| rh_superiorparietal_volume | -0.039 | -0.154 | 0.075 | -0.676 | 288.766 | 0.500 | 0.749 | 0.277 |
| rh_superiortemporal_volume | -0.139 | -0.267 | -0.012 | -2.138 | 278.523 | 0.033 | 0.196 | 0.163 |
| rh_supramarginal_volume | -0.079 | -0.201 | 0.044 | -1.257 | 282.031 | 0.210 | 0.523 | 0.187 |
| rh_frontalpole_volume | -0.026 | -0.156 | 0.104 | -0.392 | 267.326 | 0.696 | 0.781 | 0.066 |
| rh_temporalpole_volume | -0.032 | -0.162 | 0.098 | -0.475 | 269.573 | 0.635 | 0.769 | 0.075 |
| rh_transversetemporal_volume | -0.085 | -0.222 | 0.051 | -1.223 | 282.138 | 0.222 | 0.523 | 0.059 |
| rh_insula_volume | -0.159 | -0.286 | -0.033 | -2.480 | 287.569 | 0.014 | 0.125 | 0.204 |
| Left.Thalamus | -0.038 | -0.141 | 0.066 | -0.714 | 279.231 | 0.476 | 0.749 | 0.330 |
| Left.Caudate | -0.093 | -0.219 | 0.034 | -1.436 | 275.028 | 0.152 | 0.481 | 0.159 |
| Left.Putamen | -0.020 | -0.142 | 0.101 | -0.329 | 284.936 | 0.742 | 0.822 | 0.235 |
| Left.Pallidum | -0.057 | -0.167 | 0.054 | -1.006 | 279.207 | 0.315 | 0.630 | 0.260 |
| Left.Hippocampus | -0.066 | -0.179 | 0.047 | -1.145 | 274.179 | 0.253 | 0.543 | 0.228 |
| Left.Amygdala | -0.036 | -0.141 | 0.069 | -0.671 | 271.579 | 0.503 | 0.749 | 0.118 |
| Left.Accumbens.area | -0.064 | -0.197 | 0.068 | -0.948 | 272.634 | 0.344 | 0.641 | 0.067 |
| Right.Thalamus | -0.007 | -0.117 | 0.103 | -0.122 | 276.252 | 0.903 | 0.938 | 0.305 |
| Right.Caudate | -0.145 | -0.268 | -0.022 | -2.315 | 268.371 | 0.021 | 0.139 | 0.187 |
| Right.Putamen | -0.051 | -0.173 | 0.072 | -0.808 | 287.026 | 0.419 | 0.688 | 0.230 |
| Right.Pallidum | 0.023 | -0.082 | 0.128 | 0.432 | 282.074 | 0.666 | 0.769 | 0.229 |
| Right.Hippocampus | 0.068 | -0.050 | 0.186 | 1.133 | 282.359 | 0.258 | 0.543 | 0.213 |
| Right.Amygdala | -0.031 | -0.137 | 0.075 | -0.576 | 275.302 | 0.565 | 0.769 | 0.231 |
| Right.Accumbens.area | -0.032 | -0.155 | 0.091 | -0.510 | 280.490 | 0.610 | 0.769 | 0.185 |

ROI = Regions of interest

FDR = false discovery rate

**Table S9** **The results of the difference between ADHD and TD by using TS-corrected data**

|  |  | Confidence interval | |  |  |  |  |  |
| --- | --- | --- | --- | --- | --- | --- | --- | --- |
| ROI | beta | 2.50% | 97.50% | t value | d.f. | p | FDR p | r2 |
| lh_bankssts_volume | -0.120 | -0.252 | 0.011 | -1.798 | 280.704 | 0.073 | 0.333 | 0.098 |
| lh_caudalanteriorcingulate_volume | 0.036 | -0.099 | 0.171 | 0.526 | 299.893 | 0.599 | 0.862 | 0.055 |
| lh_caudalmiddlefrontal_volume | -0.039 | -0.164 | 0.085 | -0.622 | 292.600 | 0.535 | 0.827 | 0.159 |
| lh_cuneus_volume | 0.074 | -0.057 | 0.205 | 1.107 | 274.942 | 0.269 | 0.587 | 0.114 |
| lh_entorhinal_volume | -0.023 | -0.156 | 0.111 | -0.332 | 271.829 | 0.740 | 0.882 | 0.039 |
| lh_fusiform_volume | -0.102 | -0.219 | 0.015 | -1.715 | 284.363 | 0.087 | 0.358 | 0.265 |
| lh_inferiorparietal_volume | -0.028 | -0.147 | 0.092 | -0.450 | 280.870 | 0.653 | 0.867 | 0.204 |
| lh_inferiortemporal_volume | -0.149 | -0.262 | -0.035 | -2.568 | 293.392 | 0.011 | 0.110 | 0.289 |
| lh_isthmuscingulate_volume | -0.049 | -0.175 | 0.077 | -0.760 | 281.168 | 0.448 | 0.750 | 0.172 |
| lh_lateraloccipital_volume | 0.063 | -0.056 | 0.183 | 1.037 | 284.875 | 0.301 | 0.587 | 0.215 |
| lh_lateralorbitofrontal_volume | -0.235 | -0.341 | -0.130 | -4.381 | 282.921 | 0.000 | 0.001 | 0.371 |
| lh_lingual_volume | 0.006 | -0.124 | 0.136 | 0.088 | 287.015 | 0.930 | 0.949 | 0.129 |
| lh_medialorbitofrontal_volume | -0.024 | -0.139 | 0.090 | -0.416 | 281.846 | 0.678 | 0.867 | 0.290 |
| lh_middletemporal_volume | -0.151 | -0.265 | -0.037 | -2.605 | 281.383 | 0.010 | 0.110 | 0.279 |
| lh_parahippocampal_volume | -0.098 | -0.231 | 0.035 | -1.446 | 286.167 | 0.149 | 0.489 | 0.088 |
| lh_paracentral_volume | -0.006 | -0.130 | 0.119 | -0.092 | 288.577 | 0.926 | 0.949 | 0.170 |
| lh_parsopercularis_volume | -0.052 | -0.182 | 0.079 | -0.776 | 283.645 | 0.438 | 0.749 | 0.089 |
| lh_parsorbitalis_volume | -0.129 | -0.253 | -0.005 | -2.039 | 288.071 | 0.042 | 0.234 | 0.167 |
| lh_parstriangularis_volume | -0.073 | -0.199 | 0.053 | -1.130 | 282.002 | 0.259 | 0.587 | 0.112 |
| lh_pericalcarine_volume | 0.004 | -0.125 | 0.133 | 0.061 | 282.263 | 0.951 | 0.951 | 0.130 |
| lh_postcentral_volume | -0.065 | -0.187 | 0.057 | -1.046 | 287.703 | 0.296 | 0.587 | 0.195 |
| lh_posteriorcingulate_volume | -0.013 | -0.135 | 0.110 | -0.199 | 284.288 | 0.842 | 0.933 | 0.217 |
| lh_precentral_volume | -0.087 | -0.204 | 0.030 | -1.460 | 286.125 | 0.145 | 0.489 | 0.252 |
| lh_precuneus_volume | -0.142 | -0.249 | -0.036 | -2.629 | 284.721 | 0.009 | 0.110 | 0.350 |
| lh_rostralanteriorcingulate_volume | -0.025 | -0.145 | 0.094 | -0.415 | 287.824 | 0.679 | 0.867 | 0.204 |
| lh_rostralmiddlefrontal_volume | -0.051 | -0.174 | 0.072 | -0.815 | 283.211 | 0.416 | 0.734 | 0.157 |
| lh_superiorfrontal_volume | -0.005 | -0.118 | 0.107 | -0.096 | 289.261 | 0.924 | 0.949 | 0.312 |
| lh_superiorparietal_volume | -0.030 | -0.150 | 0.090 | -0.496 | 276.690 | 0.620 | 0.862 | 0.205 |
| lh_superiortemporal_volume | -0.086 | -0.212 | 0.040 | -1.335 | 283.641 | 0.183 | 0.499 | 0.169 |
| lh_supramarginal_volume | -0.140 | -0.262 | -0.018 | -2.257 | 279.536 | 0.025 | 0.185 | 0.205 |
| lh_frontalpole_volume | -0.019 | -0.146 | 0.107 | -0.301 | 273.866 | 0.763 | 0.882 | 0.105 |
| lh_temporalpole_volume | -0.112 | -0.237 | 0.012 | -1.764 | 275.478 | 0.079 | 0.340 | 0.115 |
| lh_transversetemporal_volume | -0.133 | -0.262 | -0.003 | -2.006 | 299.066 | 0.046 | 0.234 | 0.084 |
| lh_insula_volume | -0.149 | -0.268 | -0.030 | -2.456 | 273.740 | 0.015 | 0.134 | 0.255 |
| rh_bankssts_volume | -0.095 | -0.225 | 0.035 | -1.426 | 288.569 | 0.155 | 0.489 | 0.093 |
| rh_caudalanteriorcingulate_volume | -0.012 | -0.139 | 0.115 | -0.184 | 299.693 | 0.854 | 0.933 | 0.088 |
| rh_caudalmiddlefrontal_volume | 0.054 | -0.075 | 0.184 | 0.820 | 282.631 | 0.413 | 0.734 | 0.122 |
| rh_cuneus_volume | -0.024 | -0.145 | 0.097 | -0.390 | 285.338 | 0.697 | 0.867 | 0.224 |
| rh_entorhinal_volume | -0.010 | -0.142 | 0.122 | -0.142 | 263.808 | 0.887 | 0.949 | 0.057 |
| rh_fusiform_volume | -0.074 | -0.189 | 0.042 | -1.249 | 274.042 | 0.213 | 0.545 | 0.256 |
| rh_inferiorparietal_volume | -0.067 | -0.186 | 0.053 | -1.091 | 286.039 | 0.276 | 0.587 | 0.231 |
| rh_inferiortemporal_volume | -0.039 | -0.157 | 0.078 | -0.657 | 267.112 | 0.512 | 0.823 | 0.245 |
| rh_isthmuscingulate_volume | -0.005 | -0.133 | 0.123 | -0.079 | 283.530 | 0.937 | 0.949 | 0.134 |
| rh_lateraloccipital_volume | 0.053 | -0.075 | 0.180 | 0.806 | 277.113 | 0.421 | 0.734 | 0.159 |
| rh_lateralorbitofrontal_volume | -0.199 | -0.307 | -0.091 | -3.606 | 279.724 | 0.000 | 0.010 | 0.367 |
| rh_lingual_volume | -0.033 | -0.161 | 0.094 | -0.517 | 281.938 | 0.606 | 0.862 | 0.158 |
| rh_medialorbitofrontal_volume | -0.086 | -0.202 | 0.029 | -1.470 | 283.924 | 0.143 | 0.489 | 0.302 |
| rh_middletemporal_volume | -0.267 | -0.382 | -0.152 | -4.559 | 273.118 | 0.000 | 0.001 | 0.274 |
| rh_parahippocampal_volume | -0.088 | -0.219 | 0.043 | -1.318 | 288.554 | 0.189 | 0.499 | 0.058 |
| rh_paracentral_volume | -0.117 | -0.240 | 0.007 | -1.849 | 282.047 | 0.066 | 0.316 | 0.193 |
| rh_parsopercularis_volume | -0.097 | -0.226 | 0.032 | -1.469 | 285.511 | 0.143 | 0.489 | 0.111 |
| rh_parsorbitalis_volume | -0.186 | -0.312 | -0.061 | -2.907 | 278.598 | 0.004 | 0.081 | 0.163 |
| rh_parstriangularis_volume | -0.134 | -0.264 | -0.004 | -2.027 | 273.450 | 0.044 | 0.234 | 0.108 |
| rh_pericalcarine_volume | -0.062 | -0.184 | 0.060 | -0.993 | 286.184 | 0.322 | 0.599 | 0.190 |
| rh_postcentral_volume | -0.031 | -0.152 | 0.090 | -0.503 | 286.209 | 0.616 | 0.862 | 0.232 |
| rh_posteriorcingulate_volume | -0.021 | -0.145 | 0.103 | -0.327 | 280.814 | 0.744 | 0.882 | 0.213 |
| rh_precentral_volume | 0.018 | -0.107 | 0.143 | 0.279 | 286.466 | 0.780 | 0.889 | 0.167 |
| rh_precuneus_volume | -0.067 | -0.176 | 0.041 | -1.216 | 276.513 | 0.225 | 0.559 | 0.354 |
| rh_rostralanteriorcingulate_volume | -0.084 | -0.207 | 0.038 | -1.347 | 285.841 | 0.179 | 0.499 | 0.172 |
| rh_rostralmiddlefrontal_volume | -0.175 | -0.296 | -0.053 | -2.817 | 275.836 | 0.005 | 0.085 | 0.192 |
| rh_superiorfrontal_volume | -0.083 | -0.201 | 0.036 | -1.361 | 292.195 | 0.175 | 0.499 | 0.241 |
| rh_superiorparietal_volume | -0.033 | -0.148 | 0.082 | -0.559 | 286.221 | 0.577 | 0.860 | 0.270 |
| rh_superiortemporal_volume | -0.135 | -0.262 | -0.009 | -2.096 | 279.869 | 0.037 | 0.233 | 0.174 |
| rh_supramarginal_volume | -0.074 | -0.199 | 0.050 | -1.168 | 280.599 | 0.244 | 0.587 | 0.175 |
| rh_frontalpole_volume | -0.026 | -0.157 | 0.105 | -0.389 | 270.568 | 0.698 | 0.867 | 0.074 |
| rh_temporalpole_volume | -0.021 | -0.151 | 0.110 | -0.313 | 268.810 | 0.755 | 0.882 | 0.064 |
| rh_transversetemporal_volume | -0.079 | -0.215 | 0.057 | -1.139 | 286.702 | 0.255 | 0.587 | 0.060 |
| rh_insula_volume | -0.135 | -0.260 | -0.010 | -2.121 | 286.045 | 0.035 | 0.233 | 0.218 |
| Left.Thalamus | -0.038 | -0.145 | 0.070 | -0.686 | 283.576 | 0.493 | 0.809 | 0.359 |
| Left.Caudate | -0.092 | -0.218 | 0.034 | -1.431 | 277.939 | 0.153 | 0.489 | 0.159 |
| Left.Putamen | -0.023 | -0.146 | 0.100 | -0.368 | 288.611 | 0.713 | 0.873 | 0.232 |
| Left.Pallidum | -0.059 | -0.174 | 0.056 | -1.011 | 282.665 | 0.313 | 0.596 | 0.290 |
| Left.Hippocampus | -0.064 | -0.184 | 0.057 | -1.038 | 282.087 | 0.300 | 0.587 | 0.244 |
| Left.Amygdala | -0.036 | -0.158 | 0.086 | -0.579 | 280.611 | 0.563 | 0.855 | 0.227 |
| Left.Accumbens.area | -0.071 | -0.205 | 0.062 | -1.046 | 277.941 | 0.296 | 0.587 | 0.069 |
| Right.Thalamus | -0.006 | -0.117 | 0.105 | -0.107 | 282.204 | 0.915 | 0.949 | 0.322 |
| Right.Caudate | -0.148 | -0.272 | -0.025 | -2.359 | 274.164 | 0.019 | 0.156 | 0.183 |
| Right.Putamen | -0.039 | -0.162 | 0.084 | -0.622 | 287.316 | 0.535 | 0.827 | 0.220 |
| Right.Pallidum | 0.024 | -0.090 | 0.138 | 0.420 | 287.071 | 0.675 | 0.867 | 0.295 |
| Right.Hippocampus | 0.084 | -0.037 | 0.206 | 1.364 | 288.397 | 0.174 | 0.499 | 0.214 |
| Right.Amygdala | -0.011 | -0.129 | 0.107 | -0.188 | 280.607 | 0.851 | 0.933 | 0.267 |
| Right.Accumbens.area | -0.029 | -0.153 | 0.095 | -0.464 | 282.381 | 0.643 | 0.867 | 0.180 |

ROI = Regions of interest

FDR = false discovery rate

**Table S10** **The results of the difference between ADHD and TD by using ComBat-corrected data**

|  |  | Confidence interval | |  |  |  |  |  |
| --- | --- | --- | --- | --- | --- | --- | --- | --- |
| ROI | beta | 2.50% | 97.50% | t value | d.f. | p | FDR p | r2 |
| lh_bankssts_volume | -0.134 | -0.265 | -0.002 | -1.986 | 281.981 | 0.048 | 0.207 | 0.087 |
| lh_caudalanteriorcingulate_volume | -0.022 | -0.156 | 0.113 | -0.317 | 302.703 | 0.751 | 0.811 | 0.055 |
| lh_caudalmiddlefrontal_volume | -0.053 | -0.178 | 0.073 | -0.818 | 294.283 | 0.414 | 0.606 | 0.146 |
| lh_cuneus_volume | 0.092 | -0.036 | 0.221 | 1.404 | 277.566 | 0.161 | 0.409 | 0.159 |
| lh_entorhinal_volume | -0.035 | -0.170 | 0.101 | -0.504 | 274.119 | 0.615 | 0.710 | 0.021 |
| lh_fusiform_volume | -0.122 | -0.238 | -0.006 | -2.053 | 284.757 | 0.041 | 0.187 | 0.274 |
| lh_inferiorparietal_volume | -0.041 | -0.158 | 0.076 | -0.688 | 283.780 | 0.492 | 0.673 | 0.242 |
| lh_inferiortemporal_volume | -0.169 | -0.282 | -0.057 | -2.952 | 293.589 | 0.003 | 0.037 | 0.299 |
| lh_isthmuscingulate_volume | -0.065 | -0.190 | 0.061 | -1.008 | 283.029 | 0.314 | 0.535 | 0.174 |
| lh_lateraloccipital_volume | 0.080 | -0.037 | 0.197 | 1.338 | 284.831 | 0.182 | 0.409 | 0.262 |
| lh_lateralorbitofrontal_volume | -0.246 | -0.353 | -0.139 | -4.499 | 270.864 | 0.000 | 0.000 | 0.337 |
| lh_lingual_volume | 0.000 | -0.128 | 0.127 | -0.003 | 286.993 | 0.997 | 0.997 | 0.166 |
| lh_medialorbitofrontal_volume | -0.036 | -0.151 | 0.079 | -0.615 | 284.080 | 0.539 | 0.691 | 0.297 |
| lh_middletemporal_volume | -0.179 | -0.292 | -0.065 | -3.083 | 279.280 | 0.002 | 0.031 | 0.275 |
| lh_parahippocampal_volume | -0.083 | -0.216 | 0.051 | -1.214 | 286.441 | 0.226 | 0.430 | 0.082 |
| lh_paracentral_volume | -0.004 | -0.128 | 0.120 | -0.066 | 290.942 | 0.947 | 0.959 | 0.185 |
| lh_parsopercularis_volume | -0.082 | -0.214 | 0.049 | -1.232 | 284.586 | 0.219 | 0.427 | 0.063 |
| lh_parsorbitalis_volume | -0.139 | -0.264 | -0.013 | -2.168 | 281.660 | 0.031 | 0.157 | 0.143 |
| lh_parstriangularis_volume | -0.095 | -0.222 | 0.031 | -1.476 | 281.723 | 0.141 | 0.409 | 0.098 |
| lh_pericalcarine_volume | 0.021 | -0.105 | 0.148 | 0.328 | 283.352 | 0.743 | 0.811 | 0.170 |
| lh_postcentral_volume | -0.061 | -0.181 | 0.060 | -0.989 | 291.644 | 0.323 | 0.535 | 0.212 |
| lh_posteriorcingulate_volume | -0.053 | -0.179 | 0.072 | -0.831 | 284.054 | 0.407 | 0.606 | 0.179 |
| lh_precentral_volume | -0.083 | -0.201 | 0.034 | -1.393 | 287.049 | 0.165 | 0.409 | 0.252 |
| lh_precuneus_volume | -0.155 | -0.261 | -0.049 | -2.873 | 287.830 | 0.004 | 0.037 | 0.350 |
| lh_rostralanteriorcingulate_volume | -0.058 | -0.182 | 0.066 | -0.921 | 288.959 | 0.358 | 0.564 | 0.164 |
| lh_rostralmiddlefrontal_volume | -0.079 | -0.202 | 0.044 | -1.263 | 280.627 | 0.208 | 0.427 | 0.156 |
| lh_superiorfrontal_volume | -0.031 | -0.144 | 0.082 | -0.536 | 289.241 | 0.592 | 0.703 | 0.295 |
| lh_superiorparietal_volume | -0.041 | -0.161 | 0.080 | -0.662 | 278.325 | 0.508 | 0.681 | 0.207 |
| lh_superiortemporal_volume | -0.096 | -0.224 | 0.031 | -1.482 | 282.489 | 0.140 | 0.409 | 0.164 |
| lh_supramarginal_volume | -0.149 | -0.272 | -0.027 | -2.395 | 280.345 | 0.017 | 0.107 | 0.206 |
| lh_frontalpole_volume | -0.047 | -0.176 | 0.083 | -0.705 | 270.099 | 0.481 | 0.669 | 0.083 |
| lh_temporalpole_volume | -0.123 | -0.250 | 0.004 | -1.894 | 274.428 | 0.059 | 0.243 | 0.089 |
| lh_transversetemporal_volume | -0.158 | -0.289 | -0.026 | -2.355 | 301.337 | 0.019 | 0.107 | 0.075 |
| lh_insula_volume | -0.180 | -0.303 | -0.057 | -2.864 | 273.347 | 0.005 | 0.037 | 0.211 |
| rh_bankssts_volume | -0.094 | -0.226 | 0.037 | -1.412 | 289.859 | 0.159 | 0.409 | 0.084 |
| rh_caudalanteriorcingulate_volume | -0.034 | -0.161 | 0.093 | -0.525 | 300.712 | 0.600 | 0.703 | 0.082 |
| rh_caudalmiddlefrontal_volume | 0.035 | -0.095 | 0.165 | 0.526 | 282.256 | 0.599 | 0.703 | 0.113 |
| rh_cuneus_volume | -0.024 | -0.143 | 0.095 | -0.396 | 287.198 | 0.693 | 0.778 | 0.249 |
| rh_entorhinal_volume | -0.011 | -0.143 | 0.121 | -0.164 | 262.305 | 0.870 | 0.903 | 0.057 |
| rh_fusiform_volume | -0.088 | -0.202 | 0.027 | -1.494 | 273.952 | 0.136 | 0.409 | 0.269 |
| rh_inferiorparietal_volume | -0.081 | -0.198 | 0.036 | -1.356 | 289.509 | 0.176 | 0.409 | 0.260 |
| rh_inferiortemporal_volume | -0.068 | -0.185 | 0.050 | -1.130 | 265.237 | 0.259 | 0.482 | 0.243 |
| rh_isthmuscingulate_volume | -0.008 | -0.134 | 0.118 | -0.126 | 283.848 | 0.900 | 0.922 | 0.157 |
| rh_lateraloccipital_volume | 0.054 | -0.072 | 0.180 | 0.844 | 279.958 | 0.400 | 0.606 | 0.184 |
| rh_lateralorbitofrontal_volume | -0.219 | -0.325 | -0.113 | -4.031 | 278.066 | 0.000 | 0.002 | 0.380 |
| rh_lingual_volume | -0.036 | -0.161 | 0.089 | -0.565 | 281.930 | 0.573 | 0.703 | 0.187 |
| rh_medialorbitofrontal_volume | -0.107 | -0.224 | 0.011 | -1.782 | 284.545 | 0.076 | 0.270 | 0.284 |
| rh_middletemporal_volume | -0.277 | -0.391 | -0.162 | -4.730 | 267.433 | 0.000 | 0.000 | 0.278 |
| rh_parahippocampal_volume | -0.088 | -0.219 | 0.042 | -1.330 | 289.702 | 0.185 | 0.409 | 0.070 |
| rh_paracentral_volume | -0.115 | -0.239 | 0.009 | -1.817 | 283.832 | 0.070 | 0.266 | 0.187 |
| rh_parsopercularis_volume | -0.143 | -0.273 | -0.013 | -2.149 | 285.081 | 0.033 | 0.157 | 0.091 |
| rh_parsorbitalis_volume | -0.209 | -0.336 | -0.083 | -3.241 | 280.784 | 0.001 | 0.027 | 0.157 |
| rh_parstriangularis_volume | -0.172 | -0.304 | -0.041 | -2.567 | 275.766 | 0.011 | 0.080 | 0.082 |
| rh_pericalcarine_volume | -0.065 | -0.186 | 0.055 | -1.062 | 287.340 | 0.289 | 0.504 | 0.215 |
| rh_postcentral_volume | -0.038 | -0.160 | 0.083 | -0.617 | 287.660 | 0.537 | 0.691 | 0.230 |
| rh_posteriorcingulate_volume | -0.031 | -0.154 | 0.092 | -0.492 | 281.213 | 0.623 | 0.710 | 0.213 |
| rh_precentral_volume | 0.023 | -0.103 | 0.148 | 0.351 | 288.781 | 0.726 | 0.804 | 0.150 |
| rh_precuneus_volume | -0.078 | -0.185 | 0.030 | -1.417 | 281.768 | 0.158 | 0.409 | 0.364 |
| rh_rostralanteriorcingulate_volume | -0.109 | -0.234 | 0.016 | -1.712 | 285.676 | 0.088 | 0.301 | 0.151 |
| rh_rostralmiddlefrontal_volume | -0.195 | -0.319 | -0.072 | -3.099 | 274.386 | 0.002 | 0.031 | 0.179 |
| rh_superiorfrontal_volume | -0.111 | -0.231 | 0.009 | -1.811 | 291.024 | 0.071 | 0.266 | 0.225 |
| rh_superiorparietal_volume | -0.038 | -0.152 | 0.076 | -0.652 | 288.250 | 0.515 | 0.681 | 0.284 |
| rh_superiortemporal_volume | -0.153 | -0.279 | -0.027 | -2.377 | 282.386 | 0.018 | 0.107 | 0.176 |
| rh_supramarginal_volume | -0.077 | -0.200 | 0.046 | -1.232 | 284.309 | 0.219 | 0.427 | 0.190 |
| rh_frontalpole_volume | -0.067 | -0.201 | 0.067 | -0.983 | 274.402 | 0.326 | 0.535 | 0.060 |
| rh_temporalpole_volume | -0.041 | -0.172 | 0.091 | -0.602 | 268.061 | 0.548 | 0.691 | 0.051 |
| rh_transversetemporal_volume | -0.087 | -0.223 | 0.049 | -1.256 | 287.021 | 0.210 | 0.427 | 0.064 |
| rh_insula_volume | -0.188 | -0.316 | -0.060 | -2.881 | 290.175 | 0.004 | 0.037 | 0.188 |
| Left.Thalamus | -0.050 | -0.157 | 0.057 | -0.909 | 284.136 | 0.364 | 0.564 | 0.362 |
| Left.Caudate | -0.092 | -0.219 | 0.034 | -1.427 | 278.655 | 0.155 | 0.409 | 0.158 |
| Left.Putamen | -0.035 | -0.157 | 0.088 | -0.557 | 288.550 | 0.578 | 0.703 | 0.237 |
| Left.Pallidum | -0.056 | -0.176 | 0.063 | -0.920 | 279.098 | 0.358 | 0.564 | 0.243 |
| Left.Hippocampus | -0.082 | -0.202 | 0.037 | -1.348 | 279.617 | 0.179 | 0.409 | 0.256 |
| Left.Amygdala | -0.070 | -0.193 | 0.053 | -1.118 | 279.356 | 0.265 | 0.482 | 0.207 |
| Left.Accumbens.area | -0.096 | -0.232 | 0.040 | -1.387 | 279.091 | 0.167 | 0.409 | 0.054 |
| Right.Thalamus | -0.010 | -0.127 | 0.106 | -0.176 | 288.225 | 0.860 | 0.903 | 0.256 |
| Right.Caudate | -0.148 | -0.272 | -0.025 | -2.348 | 271.481 | 0.020 | 0.107 | 0.183 |
| Right.Putamen | -0.070 | -0.195 | 0.056 | -1.084 | 288.462 | 0.279 | 0.498 | 0.199 |
| Right.Pallidum | 0.014 | -0.102 | 0.130 | 0.241 | 285.823 | 0.810 | 0.862 | 0.285 |
| Right.Hippocampus | 0.080 | -0.042 | 0.202 | 1.289 | 289.084 | 0.198 | 0.427 | 0.195 |
| Right.Amygdala | -0.043 | -0.159 | 0.073 | -0.719 | 281.195 | 0.473 | 0.669 | 0.291 |
| Right.Accumbens.area | -0.046 | -0.174 | 0.082 | -0.705 | 285.344 | 0.481 | 0.669 | 0.133 |

ROI = Regions of interest

FDR = false discovery rate

**Table S11** **The results of the difference between ADHD and TD by using raw data, TS-corrected data and ComBat-corrected data**

|  | Raw | | | TS | | | ComBat | | |
| --- | --- | --- | --- | --- | --- | --- | --- | --- | --- |
| ROI | *beta* | *p* | *FDR p* | *beta* | *p* | *FDR p* | *beta* | *p* | FDR *p* |
| rh_middletemporal_volume | -0.255 | <0.01 | 0.001 | -0.277 | <0.01 | <0.01 | -0.261 | <0.01 | 0.001 |
| lh_lateralorbitofrontal_volume | -0.226 | <0.01 | 0.001 | -0.246 | <0.01 | <0.01 | -0.223 | <0.01 | 0.002 |
| rh_lateralorbitofrontal_volume | -0.193 | <0.01 | 0.008 | -0.219 | <0.01 | 0.002 | -0.194 | <0.01 | 0.013 |
| rh_parsorbitalis_volume | -0.172 | 0.005 | 0.085 | -0.209 | 0.001 | 0.027 | -0.184 | 0.004 | 0.09 |
| rh_rostralmiddlefrontal_volume | -0.167 | 0.006 | 0.085 | -0.195 | 0.002 | 0.031 | -0.173 | 0.006 | 0.091 |
| lh_middletemporal_volume | -0.153 | 0.006 | 0.085 | -0.179 | 0.002 | 0.031 | -0.148 | 0.012 | 0.158 |
| lh_inferiortemporal_volume | -0.148 | 0.009 | 0.109 | -0.169 | 0.003 | 0.037 | -0.142 | 0.015 | 0.165 |
| rh_insula_volume | -0.159 | 0.014 | 0.125 | -0.188 | 0.004 | 0.037 | -0.13 | 0.016 | 0.165 |
| lh_precuneus_volume | -0.137 | 0.012 | 0.125 | -0.155 | 0.004 | 0.037 | -0.144 | 0.019 | 0.171 |
| lh_insula_volume | -0.147 | 0.017 | 0.139 | -0.18 | 0.005 | 0.037 | -0.128 | 0.045 | 0.286 |
| rh_parstriangularis_volume | -0.153 | 0.022 | 0.139 | -0.172 | 0.011 | 0.08 | -0.131 | 0.048 | 0.286 |
| lh_supramarginal_volume | -0.143 | 0.022 | 0.139 | -0.149 | 0.017 | 0.107 | -0.142 | 0.025 | 0.202 |
| rh_superiortemporal_volume | -0.139 | 0.033 | 0.196 | -0.153 | 0.018 | 0.107 | -0.13 | 0.037 | 0.276 |
| lh_transversetemporal_volume | -0.137 | 0.042 | 0.217 | -0.158 | 0.019 | 0.107 | -0.128 | 0.049 | 0.286 |
| Right.Caudate | -0.145 | 0.021 | 0.139 | -0.148 | 0.02 | 0.107 | -0.122 | 0.066 | 0.34 |
| lh_parsorbitalis_volume | -0.124 | 0.038 | 0.209 | -0.139 | 0.031 | 0.157 | -0.118 | 0.062 | 0.338 |
| rh_parsopercularis_volume | -0.111 | 0.094 | 0.365 | -0.143 | 0.033 | 0.157 | -0.097 | 0.143 | 0.559 |
| lh_fusiform_volume | -0.105 | 0.072 | 0.327 | -0.122 | 0.041 | 0.187 | -0.091 | 0.13 | 0.533 |
| lh_bankssts_volume | -0.115 | 0.085 | 0.347 | -0.134 | 0.048 | 0.207 | -0.115 | 0.086 | 0.392 |
| lh_temporalpole_volume | -0.121 | 0.056 | 0.272 | -0.123 | 0.059 | 0.243 | -0.098 | 0.124 | 0.533 |
| rh_paracentral_volume | -0.111 | 0.079 | 0.34 | -0.115 | 0.07 | 0.266 | -0.114 | 0.071 | 0.341 |
| rh_superiorfrontal_volume | -0.084 | 0.168 | 0.49 | -0.111 | 0.071 | 0.266 | -0.083 | 0.173 | 0.574 |
| rh_medialorbitofrontal_volume | -0.092 | 0.108 | 0.404 | -0.107 | 0.076 | 0.27 | -0.073 | 0.213 | 0.596 |
| rh_rostralanteriorcingulate_volume | -0.091 | 0.147 | 0.481 | -0.109 | 0.088 | 0.301 | -0.082 | 0.192 | 0.574 |
| rh_fusiform_volume | -0.084 | 0.153 | 0.481 | -0.088 | 0.136 | 0.409 | -0.079 | 0.187 | 0.574 |
| lh_superiortemporal_volume | -0.081 | 0.207 | 0.523 | -0.096 | 0.14 | 0.409 | -0.086 | 0.196 | 0.574 |
| lh_parstriangularis_volume | -0.078 | 0.229 | 0.523 | -0.095 | 0.141 | 0.409 | -0.088 | 0.191 | 0.574 |
| Left.Caudate | -0.093 | 0.152 | 0.481 | -0.092 | 0.155 | 0.409 | -0.085 | 0.186 | 0.574 |
| rh_precuneus_volume | -0.067 | 0.221 | 0.523 | -0.078 | 0.158 | 0.409 | 0.082 | 0.221 | 0.596 |
| rh_bankssts_volume | -0.081 | 0.23 | 0.523 | -0.094 | 0.159 | 0.409 | -0.077 | 0.229 | 0.596 |
| lh_cuneus_volume | 0.078 | 0.244 | 0.542 | 0.092 | 0.161 | 0.409 | -0.072 | 0.262 | 0.6 |
| lh_precentral_volume | -0.083 | 0.173 | 0.49 | -0.083 | 0.165 | 0.409 | -0.067 | 0.261 | 0.6 |
| Left.Accumbens.area | -0.064 | 0.344 | 0.641 | -0.096 | 0.167 | 0.409 | -0.068 | 0.264 | 0.6 |
| rh_inferiorparietal_volume | -0.076 | 0.197 | 0.523 | -0.081 | 0.176 | 0.409 | -0.062 | 0.262 | 0.6 |
| Left.Hippocampus | -0.066 | 0.253 | 0.543 | -0.082 | 0.179 | 0.409 | -0.068 | 0.271 | 0.6 |
| lh_lateraloccipital_volume | 0.057 | 0.334 | 0.637 | 0.08 | 0.182 | 0.409 | 0.062 | 0.308 | 0.632 |
| rh_parahippocampal_volume | -0.092 | 0.158 | 0.481 | -0.088 | 0.185 | 0.409 | -0.07 | 0.307 | 0.632 |
| Right.Hippocampus | 0.068 | 0.258 | 0.543 | 0.08 | 0.198 | 0.427 | 0.09 | 0.15 | 0.559 |
| lh_rostralmiddlefrontal_volume | -0.062 | 0.31 | 0.63 | -0.079 | 0.208 | 0.427 | -0.066 | 0.296 | 0.632 |
| rh_transversetemporal_volume | -0.085 | 0.222 | 0.523 | -0.087 | 0.21 | 0.427 | -0.067 | 0.338 | 0.659 |
| rh_supramarginal_volume | -0.079 | 0.21 | 0.523 | -0.077 | 0.219 | 0.427 | -0.055 | 0.41 | 0.742 |
| lh_parsopercularis_volume | -0.057 | 0.391 | 0.668 | -0.082 | 0.219 | 0.427 | -0.049 | 0.437 | 0.763 |
| lh_parahippocampal_volume | -0.094 | 0.155 | 0.481 | -0.083 | 0.226 | 0.43 | -0.081 | 0.233 | 0.596 |
| rh_inferiortemporal_volume | -0.043 | 0.468 | 0.749 | -0.068 | 0.259 | 0.482 | -0.042 | 0.501 | 0.805 |
| Left.Amygdala | -0.036 | 0.503 | 0.749 | -0.07 | 0.265 | 0.482 | -0.036 | 0.546 | 0.862 |
| Right.Putamen | -0.051 | 0.419 | 0.688 | -0.07 | 0.279 | 0.498 | -0.03 | 0.63 | 0.895 |
| rh_pericalcarine_volume | -0.062 | 0.327 | 0.637 | -0.065 | 0.289 | 0.504 | -0.055 | 0.375 | 0.711 |
| lh_isthmuscingulate_volume | -0.055 | 0.389 | 0.668 | -0.065 | 0.314 | 0.535 | -0.054 | 0.381 | 0.711 |
| lh_postcentral_volume | -0.058 | 0.356 | 0.649 | -0.061 | 0.323 | 0.535 | -0.047 | 0.463 | 0.784 |
| rh_frontalpole_volume | -0.026 | 0.696 | 0.781 | -0.067 | 0.326 | 0.535 | -0.026 | 0.696 | 0.895 |
| lh_rostralanteriorcingulate_volume | -0.042 | 0.492 | 0.749 | -0.058 | 0.358 | 0.564 | -0.057 | 0.331 | 0.659 |
| Left.Pallidum | -0.057 | 0.315 | 0.63 | -0.056 | 0.358 | 0.564 | -0.037 | 0.497 | 0.805 |
| Left.Thalamus | -0.038 | 0.476 | 0.749 | -0.05 | 0.364 | 0.564 | -0.026 | 0.672 | 0.895 |
| rh_lateraloccipital_volume | 0.055 | 0.389 | 0.668 | 0.054 | 0.4 | 0.606 | 0.047 | 0.469 | 0.784 |
| lh_posteriorcingulate_volume | -0.033 | 0.598 | 0.769 | -0.053 | 0.407 | 0.606 | -0.036 | 0.567 | 0.878 |
| lh_caudalmiddlefrontal_volume | -0.029 | 0.651 | 0.769 | -0.053 | 0.414 | 0.606 | -0.008 | 0.902 | 0.952 |
| Right.Amygdala | -0.031 | 0.565 | 0.769 | -0.043 | 0.473 | 0.669 | -0.021 | 0.72 | 0.895 |
| Right.Accumbens.area | -0.032 | 0.61 | 0.769 | -0.046 | 0.481 | 0.669 | -0.029 | 0.647 | 0.895 |
| lh_frontalpole_volume | -0.03 | 0.642 | 0.769 | -0.047 | 0.481 | 0.669 | -0.009 | 0.889 | 0.952 |
| lh_inferiorparietal_volume | -0.032 | 0.583 | 0.769 | -0.041 | 0.492 | 0.673 | -0.026 | 0.665 | 0.895 |
| lh_superiorparietal_volume | -0.029 | 0.632 | 0.769 | -0.041 | 0.508 | 0.681 | -0.026 | 0.665 | 0.895 |
| rh_superiorparietal_volume | -0.039 | 0.5 | 0.749 | -0.038 | 0.515 | 0.681 | -0.03 | 0.612 | 0.895 |
| rh_postcentral_volume | -0.029 | 0.645 | 0.769 | -0.038 | 0.537 | 0.691 | -0.023 | 0.711 | 0.895 |
| lh_medialorbitofrontal_volume | -0.035 | 0.531 | 0.769 | -0.036 | 0.539 | 0.691 | -0.011 | 0.849 | 0.952 |
| rh_temporalpole_volume | -0.032 | 0.635 | 0.769 | -0.041 | 0.548 | 0.691 | -0.014 | 0.839 | 0.952 |
| rh_lingual_volume | -0.034 | 0.605 | 0.769 | -0.036 | 0.573 | 0.703 | 0.054 | 0.416 | 0.742 |
| Left.Putamen | -0.02 | 0.742 | 0.822 | -0.035 | 0.578 | 0.703 | -0.023 | 0.718 | 0.895 |
| lh_superiorfrontal_volume | -0.011 | 0.849 | 0.904 | -0.031 | 0.592 | 0.703 | -0.015 | 0.809 | 0.95 |
| rh_caudalmiddlefrontal_volume | 0.055 | 0.407 | 0.681 | 0.035 | 0.599 | 0.703 | -0.008 | 0.902 | 0.952 |
| rh_caudalanteriorcingulate_volume | -0.028 | 0.658 | 0.769 | -0.034 | 0.6 | 0.703 | -0.002 | 0.968 | 0.97 |
| lh_entorhinal_volume | -0.029 | 0.661 | 0.769 | -0.035 | 0.615 | 0.71 | -0.022 | 0.749 | 0.917 |
| rh_posteriorcingulate_volume | -0.028 | 0.652 | 0.769 | -0.031 | 0.623 | 0.71 | -0.015 | 0.811 | 0.95 |
| rh_cuneus_volume | -0.026 | 0.681 | 0.776 | -0.024 | 0.693 | 0.778 | -0.015 | 0.804 | 0.95 |
| rh_precentral_volume | 0.032 | 0.619 | 0.769 | 0.023 | 0.726 | 0.804 | 0.024 | 0.703 | 0.895 |
| lh_pericalcarine_volume | 0.004 | 0.957 | 0.965 | 0.021 | 0.743 | 0.811 | 0.032 | 0.641 | 0.895 |
| lh_caudalanteriorcingulate_volume | 0.017 | 0.809 | 0.873 | -0.022 | 0.751 | 0.811 | 0.01 | 0.878 | 0.952 |
| Right.Pallidum | 0.023 | 0.666 | 0.769 | 0.014 | 0.81 | 0.862 | 0.028 | 0.631 | 0.895 |
| Right.Thalamus | -0.007 | 0.903 | 0.938 | -0.01 | 0.86 | 0.903 | -0.008 | 0.905 | 0.952 |
| rh_entorhinal_volume | -0.016 | 0.808 | 0.873 | -0.011 | 0.87 | 0.903 | -0.006 | 0.922 | 0.957 |
| rh_isthmuscingulate_volume | -0.004 | 0.953 | 0.965 | -0.008 | 0.9 | 0.922 | 0.002 | 0.97 | 0.97 |
| lh_paracentral_volume | -0.003 | 0.965 | 0.965 | -0.004 | 0.947 | 0.959 | 0.004 | 0.956 | 0.97 |
| lh_lingual_volume | 0.01 | 0.885 | 0.931 | 0 | 0.997 | 0.997 | 0.012 | 0.854 | 0.952 |

ROI = Regions of interest

FDR = false discovery rate

TS = traveling subject

**Table S12 The results of the difference between ADHD and TD by using raw data, TS-corrected data and Combat-corrected data after matching age, sex and handedness between ADHD group and TD group**

|  | Raw data | | | TS data | | | ComBat | | |
| --- | --- | --- | --- | --- | --- | --- | --- | --- | --- |
| ROI | beta | p | FDR p | beta | p | FDR p | beta | p | FDR p |
| rh_middletemporal_volume | -0.285 | 0.000 | 0.006 | -0.259 | 0.000 | 0.037 | -0.275 | 0.000 | 0.024 |
| lh_lateralorbitofrontal_volume | -0.212 | 0.001 | 0.028 | -0.183 | 0.009 | 0.100 | -0.192 | 0.004 | 0.114 |
| lh_transversetemporal_volume | -0.223 | 0.008 | 0.092 | -0.251 | 0.003 | 0.100 | -0.224 | 0.008 | 0.114 |
| lh_insula_volume | -0.200 | 0.009 | 0.092 | -0.206 | 0.008 | 0.100 | -0.202 | 0.008 | 0.114 |
| rh_superiortemporal_volume | -0.227 | 0.005 | 0.092 | -0.225 | 0.004 | 0.100 | -0.220 | 0.007 | 0.114 |
| rh_lateralorbitofrontal_volume | -0.182 | 0.005 | 0.092 | -0.164 | 0.013 | 0.128 | -0.179 | 0.008 | 0.114 |
| rh_insula_volume | -0.207 | 0.013 | 0.114 | -0.227 | 0.007 | 0.100 | -0.202 | 0.015 | 0.172 |
| lh_precuneus_volume | -0.183 | 0.009 | 0.092 | -0.188 | 0.007 | 0.100 | -0.166 | 0.021 | 0.187 |
| lh_inferiortemporal_volume | -0.150 | 0.022 | 0.167 | -0.163 | 0.016 | 0.132 | -0.160 | 0.019 | 0.187 |
| lh_middletemporal_volume | -0.179 | 0.008 | 0.092 | -0.164 | 0.022 | 0.153 | -0.152 | 0.027 | 0.223 |
| rh_parsopercularis_volume | -0.153 | 0.059 | 0.232 | -0.201 | 0.014 | 0.128 | -0.175 | 0.033 | 0.224 |
| Right.Caudate | -0.158 | 0.036 | 0.186 | -0.147 | 0.058 | 0.268 | -0.165 | 0.032 | 0.224 |
| rh_parstriangularis_volume | -0.210 | 0.014 | 0.114 | -0.185 | 0.029 | 0.186 | -0.170 | 0.047 | 0.270 |
| lh_bankssts_volume | -0.184 | 0.034 | 0.186 | -0.174 | 0.041 | 0.241 | -0.173 | 0.045 | 0.270 |
| rh_rostralmiddlefrontal_volume | -0.166 | 0.041 | 0.189 | -0.153 | 0.059 | 0.268 | -0.158 | 0.049 | 0.270 |
| rh_paracentral_volume | -0.178 | 0.027 | 0.184 | -0.145 | 0.072 | 0.268 | -0.157 | 0.055 | 0.280 |
| rh_parsorbitalis_volume | -0.155 | 0.046 | 0.189 | -0.147 | 0.067 | 0.268 | -0.148 | 0.068 | 0.310 |
| rh_bankssts_volume | -0.141 | 0.091 | 0.294 | -0.156 | 0.060 | 0.268 | -0.151 | 0.065 | 0.310 |
| lh_fusiform_volume | -0.150 | 0.035 | 0.186 | -0.162 | 0.022 | 0.153 | -0.133 | 0.073 | 0.315 |
| rh_transversetemporal_volume | -0.176 | 0.046 | 0.189 | -0.173 | 0.050 | 0.268 | -0.152 | 0.085 | 0.348 |
| lh_supramarginal_volume | -0.141 | 0.076 | 0.285 | -0.137 | 0.091 | 0.274 | -0.134 | 0.097 | 0.378 |
| Right.Putamen | -0.123 | 0.140 | 0.382 | -0.152 | 0.070 | 0.268 | -0.131 | 0.118 | 0.403 |
| rh_precuneus_volume | -0.136 | 0.046 | 0.189 | -0.123 | 0.078 | 0.274 | -0.111 | 0.118 | 0.403 |
| rh_supramarginal_volume | -0.166 | 0.030 | 0.186 | -0.111 | 0.150 | 0.370 | -0.127 | 0.110 | 0.403 |
| Left.Thalamus | -0.087 | 0.204 | 0.472 | -0.111 | 0.102 | 0.289 | -0.104 | 0.127 | 0.418 |
| Left.Accumbens.area | -0.104 | 0.213 | 0.472 | -0.146 | 0.083 | 0.274 | -0.119 | 0.159 | 0.492 |
| rh_superiorfrontal_volume | -0.086 | 0.254 | 0.510 | -0.132 | 0.085 | 0.274 | -0.104 | 0.168 | 0.492 |
| lh_lateraloccipital_volume | 0.079 | 0.278 | 0.514 | 0.090 | 0.230 | 0.495 | 0.108 | 0.163 | 0.492 |
| lh_parsopercularis_volume | -0.144 | 0.088 | 0.294 | -0.142 | 0.093 | 0.274 | -0.112 | 0.185 | 0.522 |
| lh_parahippocampal_volume | -0.136 | 0.093 | 0.294 | -0.149 | 0.067 | 0.268 | -0.102 | 0.215 | 0.568 |
| rh_medialorbitofrontal_volume | -0.101 | 0.162 | 0.430 | -0.118 | 0.106 | 0.289 | -0.086 | 0.240 | 0.568 |
| rh_rostralanteriorcingulate_volume | -0.133 | 0.089 | 0.294 | -0.117 | 0.146 | 0.370 | -0.098 | 0.218 | 0.568 |
| lh_superiorfrontal_volume | -0.111 | 0.135 | 0.382 | -0.101 | 0.174 | 0.407 | -0.086 | 0.242 | 0.568 |
| lh_parstriangularis_volume | -0.122 | 0.135 | 0.382 | -0.110 | 0.183 | 0.418 | -0.099 | 0.230 | 0.568 |
| lh_parsorbitalis_volume | -0.117 | 0.119 | 0.360 | -0.081 | 0.312 | 0.581 | -0.092 | 0.242 | 0.568 |
| lh_precentral_volume | -0.067 | 0.357 | 0.585 | -0.048 | 0.511 | 0.710 | -0.080 | 0.257 | 0.585 |
| Left.Hippocampus | -0.070 | 0.335 | 0.573 | -0.108 | 0.145 | 0.370 | -0.082 | 0.284 | 0.629 |
| rh_parahippocampal_volume | -0.112 | 0.175 | 0.449 | -0.138 | 0.094 | 0.274 | -0.082 | 0.337 | 0.633 |
| lh_rostralanteriorcingulate_volume | -0.078 | 0.287 | 0.514 | -0.106 | 0.153 | 0.370 | -0.056 | 0.446 | 0.633 |
| lh_posteriorcingulate_volume | -0.096 | 0.244 | 0.510 | -0.105 | 0.207 | 0.459 | -0.063 | 0.455 | 0.633 |
| lh_rostralmiddlefrontal_volume | -0.104 | 0.190 | 0.472 | -0.089 | 0.272 | 0.571 | -0.078 | 0.329 | 0.633 |
| rh_posteriorcingulate_volume | -0.103 | 0.197 | 0.472 | -0.084 | 0.306 | 0.581 | -0.067 | 0.420 | 0.633 |
| lh_cuneus_volume | 0.044 | 0.602 | 0.760 | 0.087 | 0.296 | 0.581 | 0.076 | 0.371 | 0.633 |
| rh_inferiorparietal_volume | -0.084 | 0.258 | 0.510 | -0.075 | 0.321 | 0.585 | -0.073 | 0.335 | 0.633 |
| lh_isthmuscingulate_volume | -0.086 | 0.295 | 0.514 | -0.078 | 0.333 | 0.594 | -0.079 | 0.332 | 0.633 |
| Right.Amygdala | -0.059 | 0.396 | 0.613 | -0.069 | 0.344 | 0.601 | -0.056 | 0.454 | 0.633 |
| rh_caudalanteriorcingulate_volume | -0.076 | 0.347 | 0.581 | -0.075 | 0.363 | 0.611 | -0.060 | 0.471 | 0.633 |
| rh_postcentral_volume | -0.099 | 0.209 | 0.472 | -0.070 | 0.376 | 0.616 | -0.066 | 0.408 | 0.633 |
| Left.Amygdala | -0.055 | 0.426 | 0.613 | -0.063 | 0.421 | 0.651 | -0.060 | 0.457 | 0.633 |
| rh_fusiform_volume | -0.049 | 0.513 | 0.690 | -0.058 | 0.421 | 0.651 | -0.065 | 0.396 | 0.633 |
| rh_inferiortemporal_volume | -0.045 | 0.534 | 0.706 | -0.059 | 0.409 | 0.651 | -0.062 | 0.407 | 0.633 |
| Left.Putamen | -0.069 | 0.415 | 0.613 | -0.061 | 0.471 | 0.681 | -0.082 | 0.331 | 0.633 |
| rh_lingual_volume | -0.056 | 0.509 | 0.690 | -0.061 | 0.461 | 0.681 | -0.062 | 0.468 | 0.633 |
| Left.Caudate | -0.086 | 0.254 | 0.510 | -0.049 | 0.529 | 0.718 | -0.066 | 0.374 | 0.633 |
| Left.Pallidum | -0.078 | 0.267 | 0.510 | -0.038 | 0.618 | 0.770 | -0.069 | 0.341 | 0.633 |
| lh_superiortemporal_volume | -0.088 | 0.288 | 0.514 | -0.044 | 0.603 | 0.770 | -0.069 | 0.395 | 0.633 |
| lh_paracentral_volume | -0.071 | 0.365 | 0.587 | -0.038 | 0.629 | 0.770 | -0.057 | 0.459 | 0.633 |
| lh_lingual_volume | 0.019 | 0.817 | 0.906 | 0.039 | 0.620 | 0.770 | 0.062 | 0.452 | 0.633 |
| lh_postcentral_volume | -0.045 | 0.568 | 0.727 | -0.033 | 0.674 | 0.801 | -0.063 | 0.418 | 0.633 |
| lh_entorhinal_volume | 0.063 | 0.396 | 0.613 | 0.027 | 0.733 | 0.847 | 0.076 | 0.349 | 0.633 |
| Right.Hippocampus | 0.052 | 0.508 | 0.690 | 0.020 | 0.798 | 0.883 | 0.062 | 0.432 | 0.633 |
| Right.Thalamus | -0.028 | 0.685 | 0.814 | -0.072 | 0.310 | 0.581 | -0.047 | 0.507 | 0.671 |
| rh_pericalcarine_volume | -0.088 | 0.265 | 0.510 | -0.080 | 0.303 | 0.581 | -0.048 | 0.548 | 0.681 |
| lh_medialorbitofrontal_volume | -0.043 | 0.546 | 0.711 | -0.066 | 0.365 | 0.611 | -0.045 | 0.540 | 0.681 |
| lh_temporalpole_volume | -0.061 | 0.426 | 0.613 | -0.055 | 0.482 | 0.681 | -0.047 | 0.543 | 0.681 |
| rh_entorhinal_volume | 0.057 | 0.489 | 0.690 | 0.059 | 0.481 | 0.681 | 0.052 | 0.531 | 0.681 |
| lh_caudalmiddlefrontal_volume | -0.021 | 0.791 | 0.900 | -0.019 | 0.808 | 0.883 | -0.039 | 0.607 | 0.743 |
| lh_pericalcarine_volume | 0.020 | 0.807 | 0.906 | 0.058 | 0.476 | 0.681 | 0.040 | 0.635 | 0.766 |
| rh_superiorparietal_volume | -0.057 | 0.420 | 0.613 | -0.045 | 0.534 | 0.718 | -0.031 | 0.679 | 0.806 |
| rh_frontalpole_volume | 0.015 | 0.859 | 0.907 | 0.016 | 0.852 | 0.907 | 0.029 | 0.739 | 0.865 |
| Right.Pallidum | -0.013 | 0.850 | 0.907 | 0.018 | 0.807 | 0.883 | -0.019 | 0.781 | 0.902 |
| Right.Accumbens.area | -0.032 | 0.684 | 0.814 | -0.038 | 0.641 | 0.773 | -0.019 | 0.818 | 0.919 |
| rh_precentral_volume | -0.006 | 0.940 | 0.952 | -0.006 | 0.935 | 0.970 | -0.019 | 0.813 | 0.919 |
| lh_inferiorparietal_volume | -0.024 | 0.740 | 0.867 | -0.025 | 0.734 | 0.847 | -0.013 | 0.867 | 0.938 |
| rh_lateraloccipital_volume | 0.024 | 0.771 | 0.890 | 0.016 | 0.849 | 0.907 | 0.014 | 0.870 | 0.938 |
| lh_frontalpole_volume | -0.017 | 0.836 | 0.907 | -0.011 | 0.898 | 0.944 | -0.015 | 0.860 | 0.938 |
| rh_temporalpole_volume | -0.002 | 0.981 | 0.981 | -0.045 | 0.568 | 0.752 | 0.010 | 0.899 | 0.957 |
| rh_cuneus_volume | -0.038 | 0.647 | 0.792 | -0.005 | 0.947 | 0.971 | 0.006 | 0.944 | 0.988 |
| lh_superiorparietal_volume | -0.007 | 0.924 | 0.947 | 0.001 | 0.990 | 0.990 | 0.005 | 0.951 | 0.988 |
| rh_isthmuscingulate_volume | -0.011 | 0.890 | 0.924 | 0.004 | 0.963 | 0.975 | 0.004 | 0.964 | 0.988 |
| lh_caudalanteriorcingulate_volume | 0.015 | 0.863 | 0.907 | -0.047 | 0.586 | 0.763 | 0.002 | 0.982 | 0.994 |
| rh_caudalmiddlefrontal_volume | 0.039 | 0.621 | 0.771 | 0.021 | 0.795 | 0.883 | 0.000 | 0.997 | 0.997 |

ROI = Regions of interest

FDR = false discovery rate

TS = traveling subject

**Figure S1** **The regions (blue) with significant differences among different MRI scanners.**


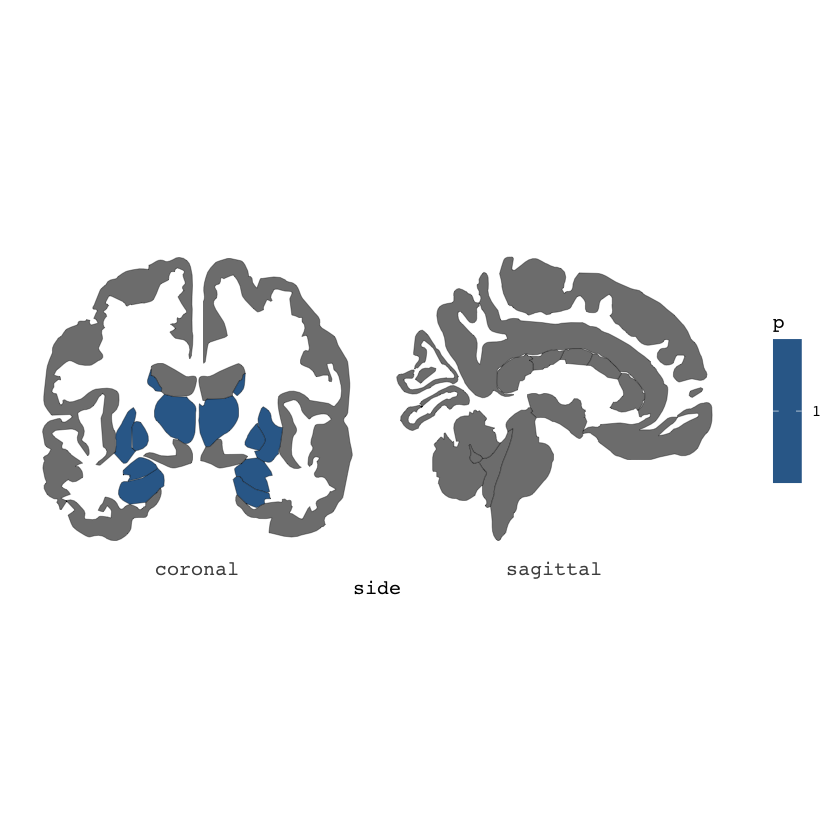

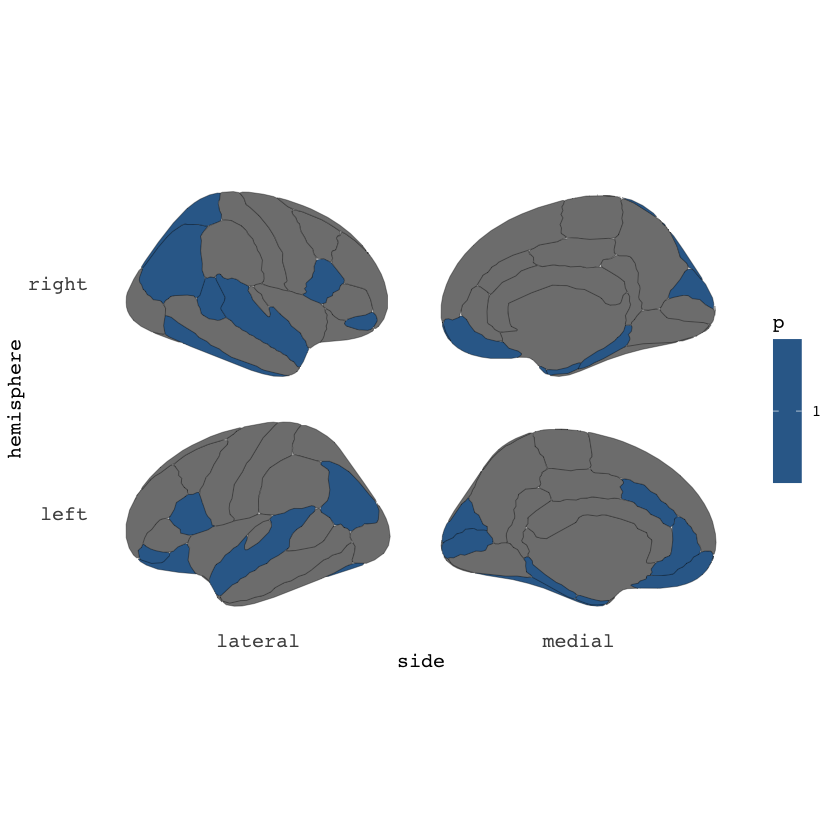


**Figure S2**


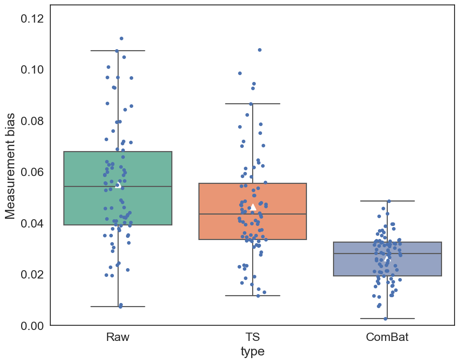

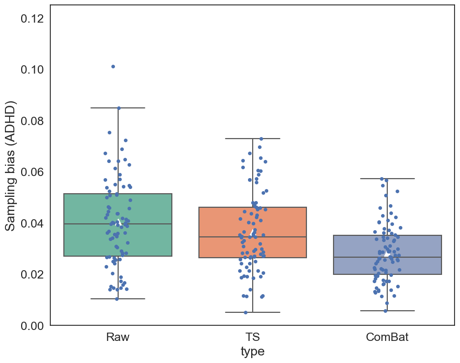

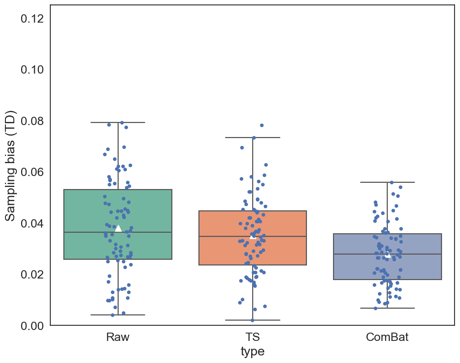


The model calculated the result of measurement bias and sampling bias with participants as a random intercept. ANOVA revealed significant differences among the data for measurement bias (F(2,243) = 42.48, p < 0.001) and sampling bias in the ADHD (F(2,243) = 18.80, p < 0.001) and TD (F(2,243) = 16.85, p < 0.001) groups. Post hoc tests indicated that the measurement bias of TS-corrected data (M = 0.043, SD = 0.021) was smaller than that of the raw data (M = 0.051, SD = 0.022) (FWE p = 0.034), but larger than that of the ComBat-corrected data (M = 0.025, SD = 0.009) (FWE p < 0.001). The measurement bias of the ComBat-corrected data was significantly smaller than that of the raw data (FWE p = 0.001). For the TD group, both TS-corrected data (M = 0.029, SD = 0.014) and raw data (M = 0.031, SD = 0.015) had a larger sampling bias than the ComBat-corrected data (M = 0.019, SD = 0.010) (TS vs. ComBat: FWE p < 0.001; raw vs. ComBat: FWE p < 0.001), with no significant difference between TS-corrected data and raw data (FWE p = 0.699). Similarly, for the ADHD group, both TS-corrected data (M = 0.031, SD = 0.013) and raw data (M = 0.034, SD = 0.015) had a larger sampling bias than the ComBat-corrected data (M = 0.022, SD = 0.009) (TS vs. ComBat: FWE p = 0.001; raw vs. ComBat: FWE p < 0.001), with no significant difference between TS-corrected data and raw data (FWE p = 0.191).

**Figure S3**


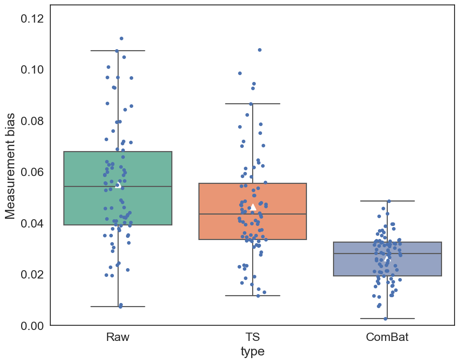

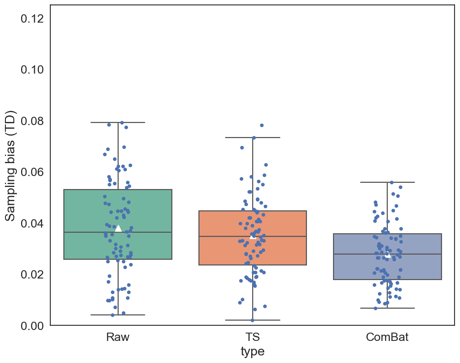

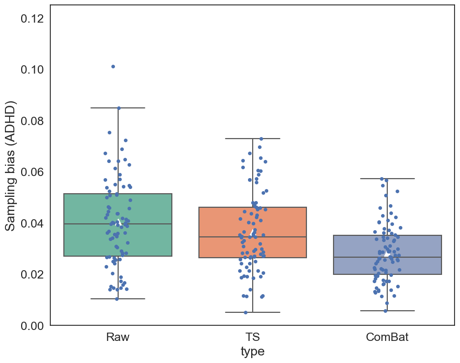


The model calculated measurement bias and sampling bias without considering participants. ANOVA results indicated significant differences in measurement bias (F(2,243) = 70.36, p < 0.001) and sampling bias in the ADHD group (F(2,243) = 20.99, p < 0.001) and TD group (F(2,243) = 17.57, p < 0.001). Post hoc tests revealed that the measurement bias of TS-corrected data (M = 0.046, SD = 0.020) was smaller than that of raw data (M = 0.055, SD = 0.023) (FWE p = 0.007) but larger than that of ComBat-corrected data (M = 0.022, SD = 0.008) (FWE p < 0.001). Measurement bias of ComBat-corrected data was significantly smaller than that of raw data (FWE p < 0.001). TS-corrected data (M = 0.033, SD = 0.018) and raw data (M = 0.035, SD = 0.018) exhibited larger sampling bias than ComBat-corrected data in the TD group (M = 0.027, SD = 0.012) (TS vs. ComBat: FWE p < 0.001; raw vs. ComBat: FWE p < 0.001), with no significant difference between TS-corrected data and raw data (FWE p = 0.619). TS-corrected data (M = 0.036, SD = 0.016) and raw data (M = 0.040, SD = 0.018) showed larger sampling bias than ComBat-corrected data in the ADHD group (M = 0.028, SD = 0.011) (TS vs. ComBat: FWE p < 0.001; raw vs. ComBat: FWE p < 0.001), with no significant difference between TS-corrected data and raw data (FWE p = 0.182).
